# Global Overview of the Use and Effectiveness of Ultraviolet C Technology in Fresh, Minimally Processed Apples and Apple Juice Over the Last 10 Years

**DOI:** 10.1002/fsn3.72316

**Published:** 2026-09-17

**Authors:** Mahyra da Paixão e Silva, Caroline Rodrigues de Azevedo, Caroline Corrêa de Souza Coelho, Juliana Pereira Rodrigues Belas, Antonio Gomes Soares, Amauri Rosenthal, Otniel Freitas‐Silva

**Affiliations:** ^1^ Departamento de Ciência e Tecnologia de Alimentos Universidade Federal Rural do Rio de Janeiro Seropédica Brazil; ^2^ Universidade Federal do Estado do Rio de Janeiro Rio de Janeiro Brazil; ^3^ Embrapa Roraima Boa Vista Brazil; ^4^ Embrapa Agroindústria de Alimentos Rio de Janeiro Brazil

**Keywords:** apple products, bibliometric analysis, microbial inactivation, non‐thermal technologies, quality preservation, UV‐C technology

## Abstract

The apple is a fruit with significant economic value and a rich nutritional profile, containing high levels of fiber, vitamins, minerals, and bioactive compounds. However, it is susceptible to chemical‐enzymatic oxidation and microbial spoilage. To extend shelf life, UV‐C radiation is a promising technology because it can achieve microbial safety without compromising post‐harvest quality. This bibliometric analysis reviewed publications from the past ten years on the use of UV‐C technology in fresh, minimally processed apples and apple juice as an alternative sanitization method. The results demonstrate the effectiveness of UV‐C, especially when combined with other technologies. Among the 52 scientific publications selected, the most notable contributions to the application of radiation in fresh apples and their derivatives came from Spain (11 articles), the United States (9 articles), and China (7 articles). Food Control was the primary scientific source of information. Overall, the results indicate that UV‐C can increase microbiological safety, extend shelf life, and preserve the sensory and nutritional qualities of apples and apple products. However, important challenges remain, such as limited radiation penetration, the lack of standardized application protocols, and the need for studies that address actual industrial‐scale processing conditions. In summary, UV‐C radiation emerges as a promising and sustainable tool for sanitizing apples and apple products, with the potential to replace or complement chlorine use. Therefore, strengthening applied research focused on industrial applications will be essential to establish this technology in the fruit and vegetable sector.

## Graphical abstract

A decade of UV‐C irradiation in Apple postharvest processing: from whole fruit to juice.

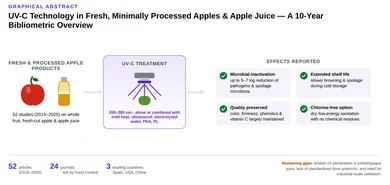

## 1 Introduction

Apples ( *Malus domestica* ) are among the most widely produced and consumed fruits worldwide, with significant economic, social, and nutritional value. They are rich in fiber, vitamins, minerals, and bioactive compounds (Arnold and Gramza‐Michałowska 2022). In 2023, global apple production exceeded 97 million tons, with major producers including China, the United States, Turkey, Poland, and India (FAOSTAT 2025). In Brazil, approximately 1.1 million tons were produced, with the Gala and Fuji varieties leading in consumer preference (ABPM 2023).

Despite their importance, the sensory and commercial quality of apples can be compromised by microbiological and physiological factors, especially during storage and post‐harvest processing (Zhu et al. 2022). Physiological processes such as enzymatic browning reduce consumer acceptability, while contamination by pathogenic microorganisms poses a risk to food safety, affecting fresh apples and other forms of the fruit, including minimally processed products, juices, and derivatives (Hasan et al. 2017).

Post‐harvest diseases in apples present a considerable challenge in the global supply chain, contributing to food losses and waste ranging from 5% to 20% of harvests (Luciano‐Rosario et al. 2025; Nicolau‐Lapeña et al. 2023). The predominant post‐harvest disease is blue mold, most often caused by species of the fungus *Penicillium*, particularly *Penicillium expansum*, which is notable for its ability to grow at low temperatures and remains a threat even in refrigerated storage (Rios de Souza et al. 2020). In addition to *Penicillium*, bacteria such as *Listeria monocytogenes* and *Escherichia coli* O157:H7 are associated with outbreaks in apples (Ho et al. 2020; Murray et al. 2018), and yeasts such as *Saccharomyces* and *Zygosaccharomyces* can grow at refrigeration temperatures (4°C), causing changes in color, texture, gas production, and off‐flavors (Graça et al. 2020; Xiang et al. 2020).

Fruit sanitization is a crucial step in post‐harvest operations, playing a key role in reducing surface microbial load and preventing foodborne illnesses. In addition to enhancing microbiological safety, this process is vital for extending shelf life and preserving the sensory and nutritional qualities of fruit (Yuan et al. 2022).

Chlorine is widely used to disinfect fruits and vegetables due to its low cost, broad availability, and proven antimicrobial efficacy. However, the formation of potentially toxic chlorinated by‐products (trihalomethanes, haloacetic acids, chloramines) and reduced effectiveness in the presence of organic matter are notable limitations, prompting the search for safer, more sustainable, and effective alternative technologies (Shin et al. 2024; Kim et al. 2021). Ultraviolet C (UV‐C) radiation has emerged as a promising alternative for sanitizing plant products, including apples and apple products (Gabriel and Ancog 2019; Tirono 2023). Its active principle is the inactivation of microorganisms by disrupting their genetic material (DNA and RNA). Because it does not generate chemical residues, UV‐C does not compromise sensory or nutritional quality as conventional chlorine‐based sanitizers do (Arcos‐Limiñana et al. 2025). Furthermore, studies indicate that UV‐C application can contribute not only to microbiological safety but also to the maintenance or even increase of bioactive compounds related to the quality and functional value of fruits (da Paixão e Silva et al. 2026; Kim et al. 2021).

Given the increasing number of studies on UV‐C radiation in apples and their by‐products, it is important to use a bibliometric approach to systematize and quantify scientific output in this area. This strategy enables an understanding of the temporal and thematic evolution of research and highlights persistent gaps, such as the lack of standardization in doses and exposure times, methodological heterogeneity among studies, and the scarcity of industrial‐scale investigations. This approach provides a comprehensive and strategic overview of the method's development, guiding future research and promoting technical and scientific advances in the use and effectiveness of UV‐C radiation in the apple production chain.

Therefore, the objective of this study was to conduct a comprehensive bibliometric review of scientific publications on the use of UV‐C radiation in sanitizing fresh and minimally processed apples, juices, and other apple‐derived products, emphasizing its effects on microbiological safety, chemical stability, and post‐harvest quality.

## 2 Methodology

This systematic review was conducted in accordance with the Preferred Reporting Items for Systematic Reviews and Meta‐Analyses (PRISMA). Systematic mapping was performed using the StArt protocol (State of the Art through Systematic Review, version 3.0.3 BETA), developed by the Software Engineering Research Laboratory (LaPES/UFSCar).

During the planning stage, the study topics and research questions were defined, along with keywords and search strings (combinations of keywords using Boolean operators). The literature search was conducted in ScienceDirect, Scopus, and Web of Science, which were selected for their comprehensive coverage of peer‐reviewed literature and relevance to the fields of food science, food processing, and applied technologies. The search results were exported in RIS format and imported into the StArt software. The inclusion and exclusion criteria for selecting studies were defined according to the main objective of this review, which was to identify the main impacts of applying UV‐C technology as a sanitizer on apples and apple products. Full search strategies with exact query strings are provided in the Supporting Information.

In the execution stage, searches were conducted using the established strings, retrieving studies from the literature. After a thorough analysis of the documents, the articles were classified into three categories: (i) accepted, (ii) rejected, or (iii) duplicates according to the criteria defined in the protocol.

After screening the accepted articles, the summarization stage involved organizing the information in Excel, enabling the consolidation of bibliometric results and the extraction of data according to the previously defined variables. This stage aimed to collect contextual information from the studies and organize it systematically to allow comparison and synthesis of findings across different works.

**FIGURE 1:**
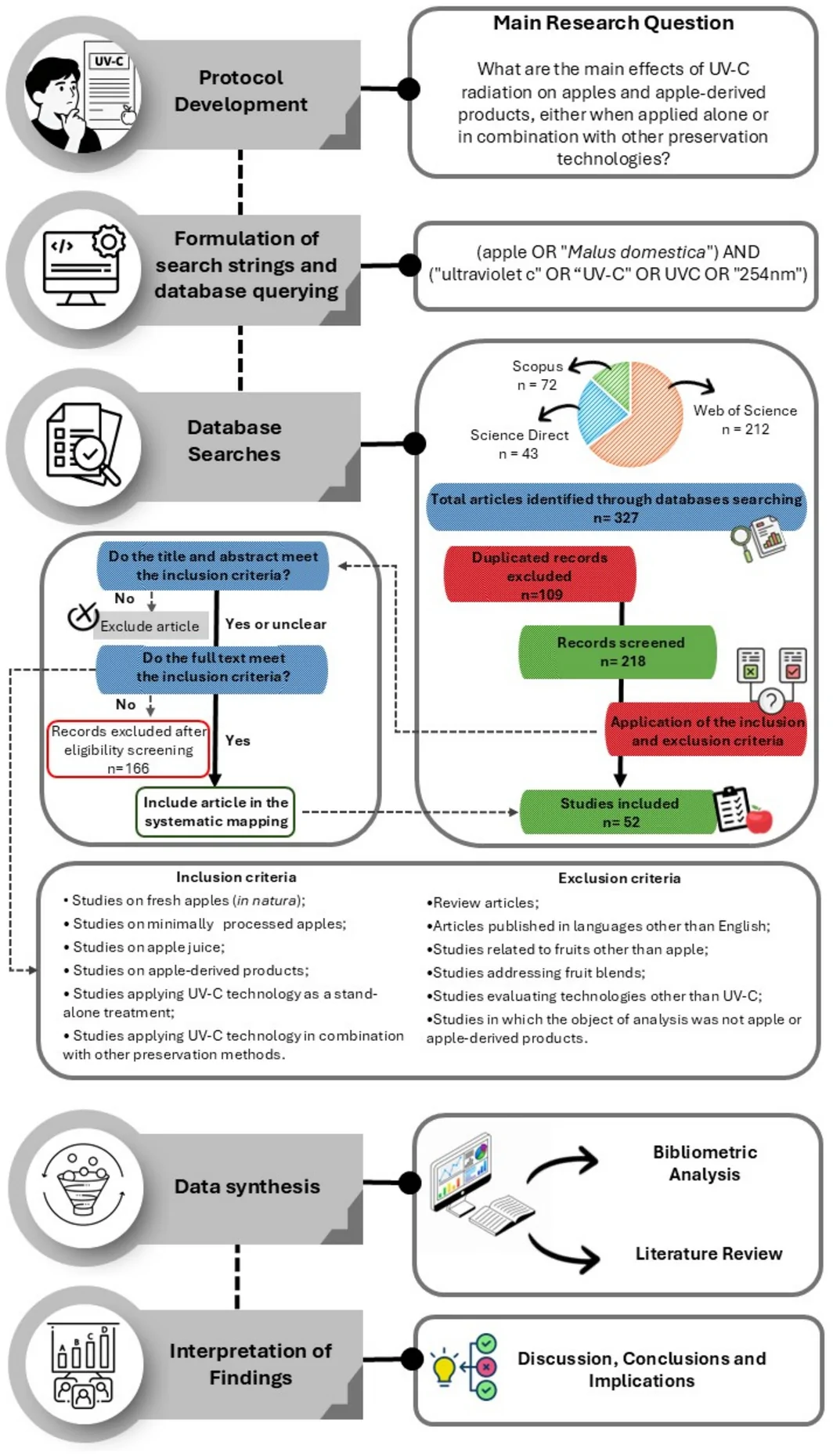
Flow diagram of the systematic review process, including search, screening, selection, and synthesis of findings.

The review process took place in June 2025. All steps described above in this bibliometric review are detailed in Figure 1. Research articles published in journals with impact factors were included if the full text was available in English, either through paid or open access and published between January 1, 2015, and June 10, 2025. After searching the databases, 327 publications were identified: 72 from Scopus, 212 from Web of Science, and 43 from ScienceDirect. Following the screening stage, 218 studies were evaluated in full. Ultimately, 52 articles that met the inclusion criteria were selected for data extraction. These studies directly address the main research question and meet the criteria defined in the protocol. Therefore, they were included in this review and are presented and discussed in the following sections.

In addition to bibliometric information, such as main journals, year of publication, and countries, contextual data were also extracted from the selected articles, including the type of product studied, specific conditions of UV‐C application, and whether the technology was applied alone or in combination with other emerging technologies (hurdle technology).

Data analysis was performed by creating graphs and figures using Google Charts and network maps with VOSviewer.

## 3 Results and Discussion

### 3.1 Bibliometric Analysis and Scientific Performance

#### 3.1.1 Publication Trends Over the Years

**FIGURE 2:**
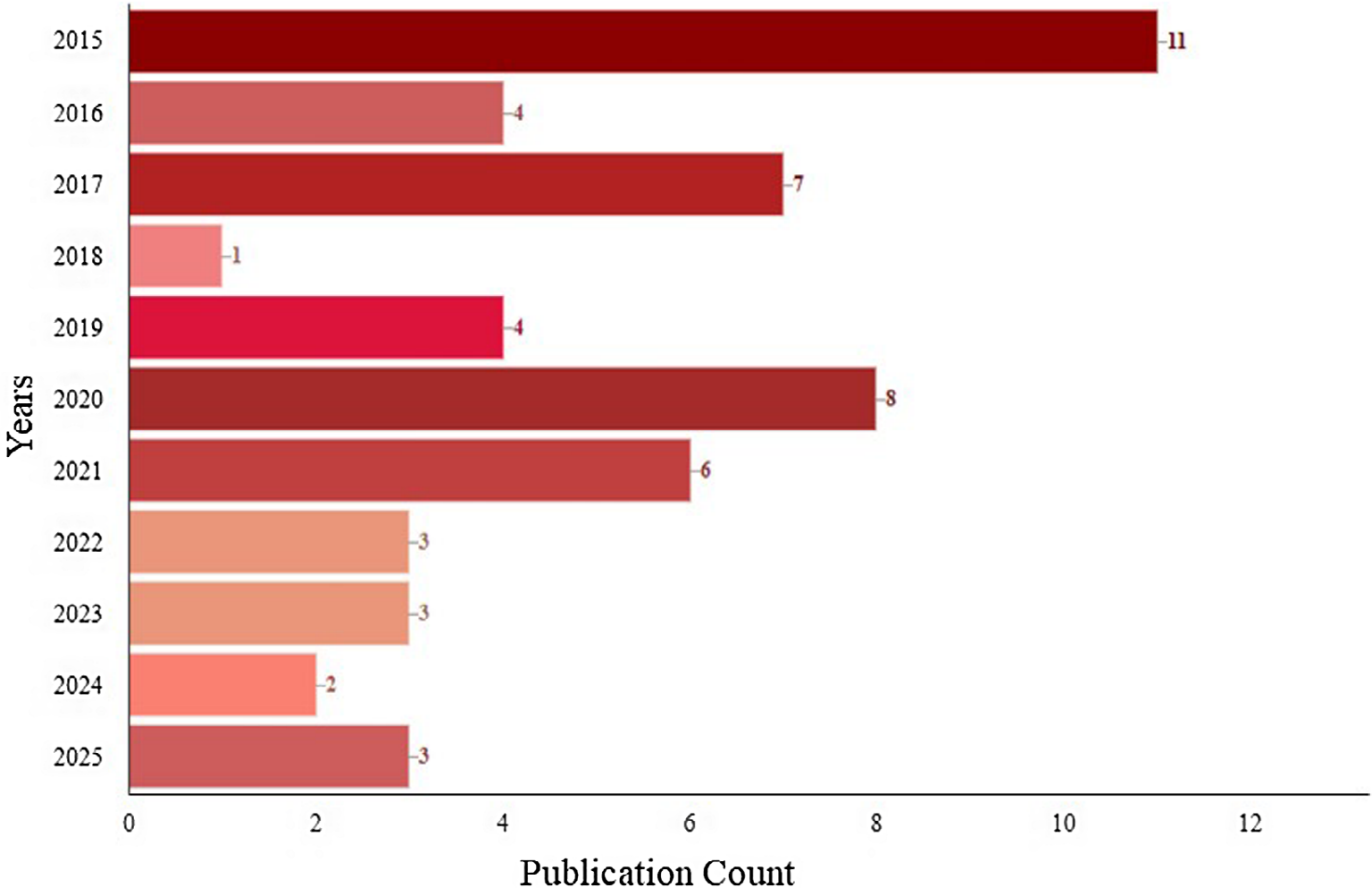
Temporal evolution of published works.

Figure 2 shows the temporal evolution of publications on the application of UV‐C radiation to fresh apples and their derivatives, such as minimally processed apples and apple juice, with a focus on the conservation and extension of shelf life for plant products. The figure indicates growing scientific interest in this topic. In 2015, 21% of the studies included in this review were published, while in 2016 and 2017, 8% and 13% of the publications were recorded, respectively. This trend reflects increasing interest in emerging technologies for processing and preserving apples and apple products. It may also be related to the need to expand microbiological control strategies, especially in response to contamination outbreaks in apple‐based products worldwide, which have increased demand for innovative methods such as UV‐C radiation.

An outbreak of listeriosis ( *L. monocytogenes* ) in 2014–2015 in the United States, associated with caramel apples, alerted the industry and research institutions to microbial risks in apple products, especially when subjected to post‐harvest treatments (Carstens et al. 2019). Several articles evaluated in this study (Tirono 2023; Nicolau‐Lapeña et al. 2022; Kim et al. 2021; Green et al. 2020; Gabriel and Ancog 2019; Murray et al. 2018; Adhikari et al. 2015; Gayán et al. 2015) investigated the effectiveness of UV‐C alone or in combination with additional barriers, such as slightly acidic electrolyzed water (SAEW) with fumaric acid (FA) and UV‐C LED in minimally processed apples (Kim et al. 2021). In fresh apples, Murray et al. (2018) evaluated a sequential strategy to inactivate *L. monocytogenes* , specifically for the production of caramel apples. The treatment combined forced‐air ozone followed by an advanced oxidation process (AOP) that integrates UV‐C, ozone, and hydrogen peroxide vapor.

The years 2020 and 2021 saw a higher volume of studies published on the topic, accounting for 15% and 12% of publications, respectively. This suggests periods of renewed interest or methodological consolidation in the application of UV‐C radiation to apples and their derivatives. The increase in studies during this time coincided with the outbreak of the COVID‐19 pandemic. During this period, concerns about food safety became more prominent, driving demand for effective antimicrobial strategies to extend food shelf life. As a result, global awareness of pathogen control and food hygiene may have contributed to the increased interest in UV‐C technology applications for fresh and processed products.

The pattern of publication peaks followed by periods of lower output, as observed in subsequent years (2023–2025), indicates intervals of reduced activity or a shift in research focus. Although UV‐C radiation is an emerging technology widely studied in various foods, this review focuses exclusively on its application in apples and apple products. Therefore, the results specifically reflect scientific interest in using UV‐C to enhance microbiological safety and extend the shelf life of fresh, minimally processed, or processed apples (juices, purees), without extrapolating to broader applications of UV‐C technology in food. The total of 52 articles identified through this methodological approach demonstrates ongoing scientific interest in UV‐C radiation for apples and their derivatives.

#### 3.1.2 Scientific Mapping Analysis

**FIGURE 3:**
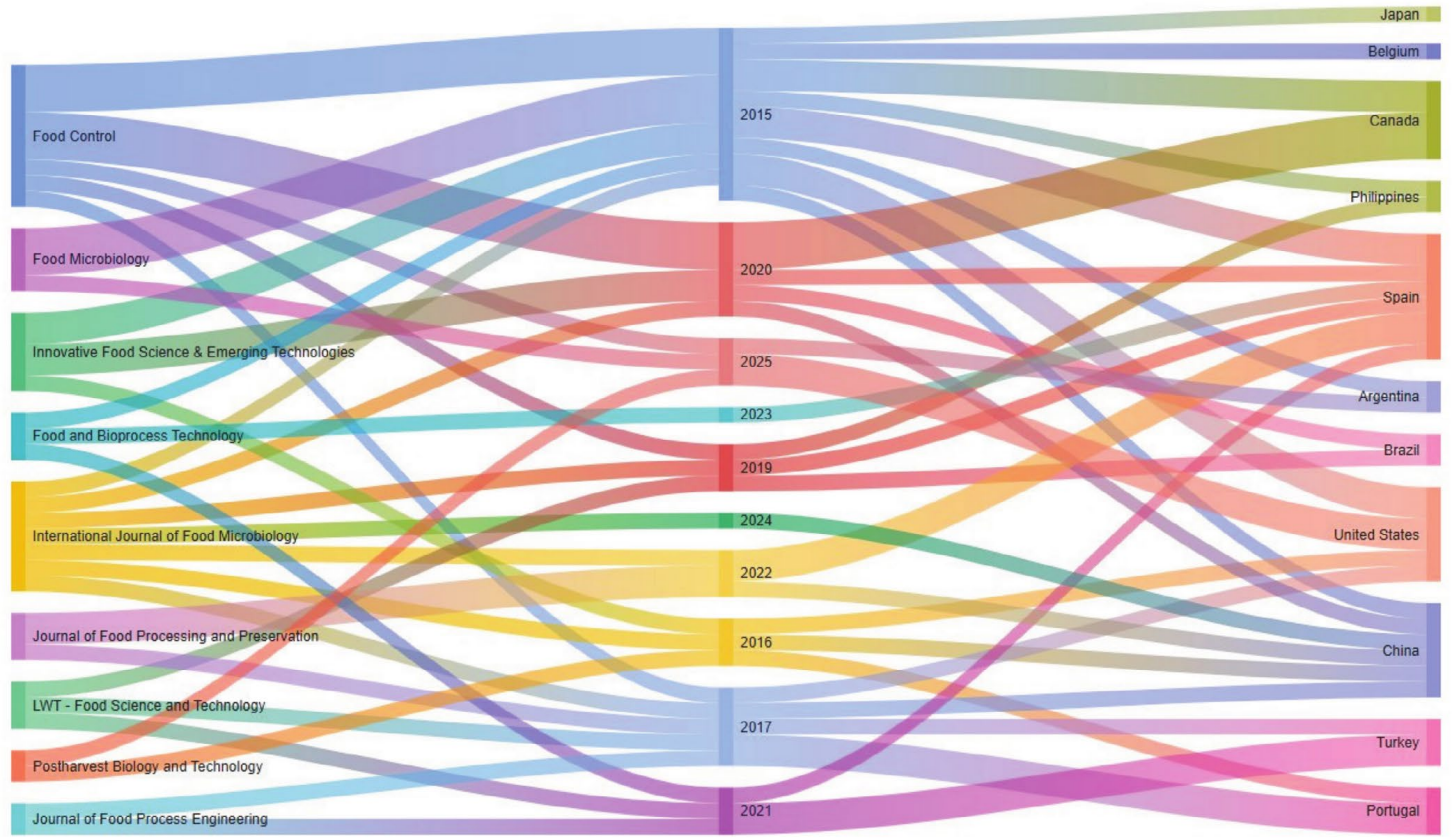
Sankey diagram showing the main journals, years of publication, and countries from major research groups.

Figure 3 presents the Sankey diagram illustrating the main journals, years of publication, and countries. The bibliometric analysis identified 52 publications on the topic, published in 24 different journals between 2015 and 2025, demonstrating widespread scientific dissemination and growing interest in the application of UV‐C radiation to apples and apple‐based products, such as juices. To highlight the most relevant journals, the nine main publication journals identified in this bibliometric study were emphasized. Among these, Food Control stood out as the leading journal publishing studies on the application of UV‐C radiation to apples, contributing nine articles on the topic, with the highest number of publications in 2015 and 2020, with three articles in each year.

The International Journal of Food Microbiology and Innovative Food Science & Emerging Technologies also contributed a substantial number of publications, with seven and five articles, respectively. The journals identified in this study cover research on food safety and quality, as well as the development of post‐harvest preventive measures—areas directly related to the use of UV‐C radiation as a non‐thermal technology for preserving and extending the shelf life of plant products.

Most publications appeared in 2015, driven by work led by Spanish and Canadian groups in journals such as Food Microbiology and Food Control. Since 2017, Brazil, Portugal, and Turkey have emerged as new scientific contributors, with a noticeable increase in publications. In 2020, the topic became more established, with expanded international collaborations. Canada, Spain, and China led in the number of articles, with a greater concentration in journals such as Food Control and Innovative Food Science & Emerging Technologies. In 2025, publications are concentrated in the United States and Argentina, in journals such as Food Control, Innovative Food Microbiology, and Postharvest Biology and Technology.

**FIGURE 4:**
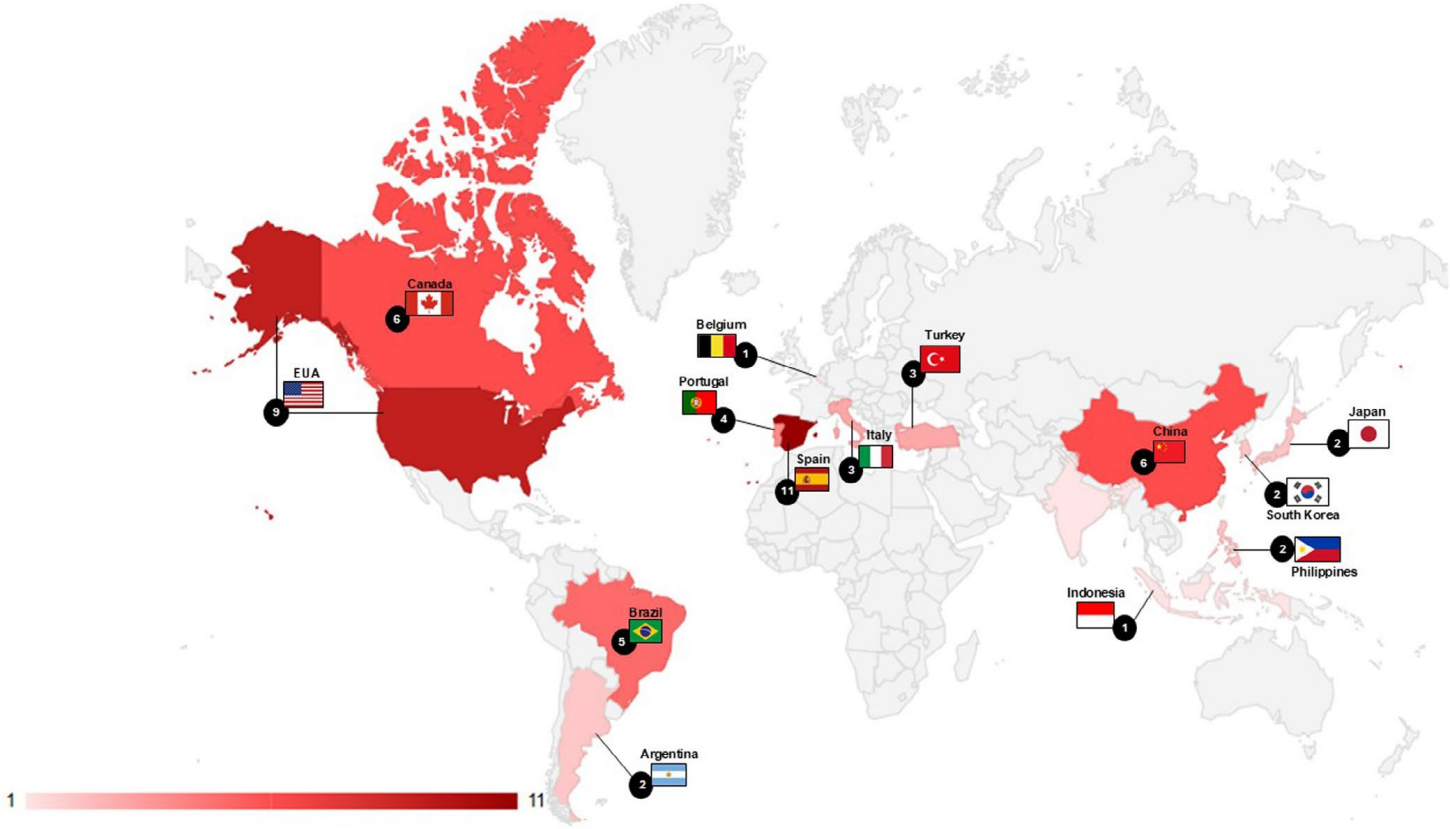
Spatial distribution of scientific publications by country. Software Google Charts.

Among the countries with the greatest scientific contributions to the application of UV‐C radiation to fresh apples and their derivatives, Spain (11 articles), the United States (9 articles), and China (7 articles) stand out (Figure 4). The prominence of China and the United States in these publications may be linked to their significant apple production, estimated at 49.6 and 5.15 million tons, respectively, in 2023 (FAOSTAT 2025), as well as advances in research on post‐harvest conservation and quality. Publications from these countries are concentrated in Postharvest Biology and Technology and the Journal of Food Processing and Preservation.

Spain produced around 528 tons of apples in the same period (FAOSTAT 2025). Although its production volume is lower, Spain has a significant share of publications on UV‐C radiation applied to apples and has the highest number of connections, particularly in the journals Innovative Food Science & Emerging Technologies and Food Control. This may be related not only to interest in fresh produce but also to Spain's notable role in research and technological innovation focused on minimal processing and the production of fruit juices and purées. As shown in this bibliographic research, articles published or co‐authored by Spanish researchers are linked to the University of Zaragoza (Aragon Agri‐Food Institute—IA2), the Autonomous University of Barcelona (CIRTTA, XaRTA, TECNIO‐CERTA, and SPTA), the University of Lleida (AGROTECNIO‐CERCA Center), and the University of Navarra (CEIT‐BRTA). These centers are part of agri‐food innovation networks with a consolidated scientific structure, which explains Spain's leadership in publications on this subject.

#### 3.1.3 Keyword Analysis

**FIGURE 5:**
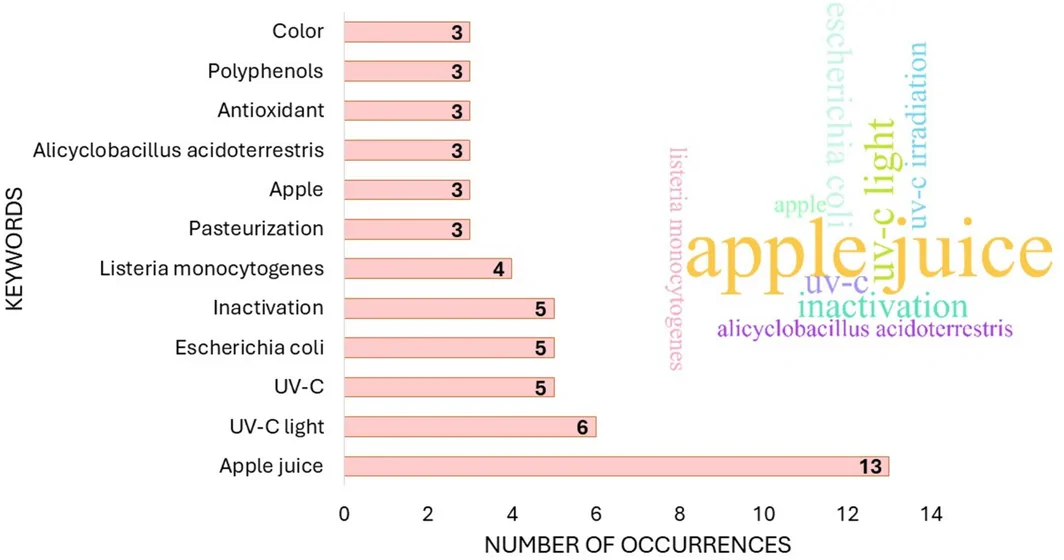
Number of keyword occurrences.

The analysis of keywords (Figure 5) showed that terms directly related to the application of UV‐C radiation in apple matrices and their derivatives were predominant. The term “apple juice” appeared most frequently (13 records), indicating that juice is the main product used in UV‐C irradiation experiments on apples.

Following this, “UV‐C light” (6 occurrences), “UV‐C,” “ *Escherichia coli* ,” and “inactivation” (5 occurrences each) demonstrate the recurring focus on microbial inactivation mechanisms and the evaluation of UV‐C radiation efficiency against different target microorganisms, including both food safety pathogens and spoilage fungi, as discussed in Section 3.4.

Terms such as “pasteurization” (3 occurrences), “Ultra‐High‐Pressure Homogenization (HHP)” (2 occurrences), and “ozone” (1 occurrence) indicate an interest in comparing different technologies for preserving the physical, chemical, and sensory properties of apples and apple products, as discussed in Section 3.5.

**FIGURE 6:**
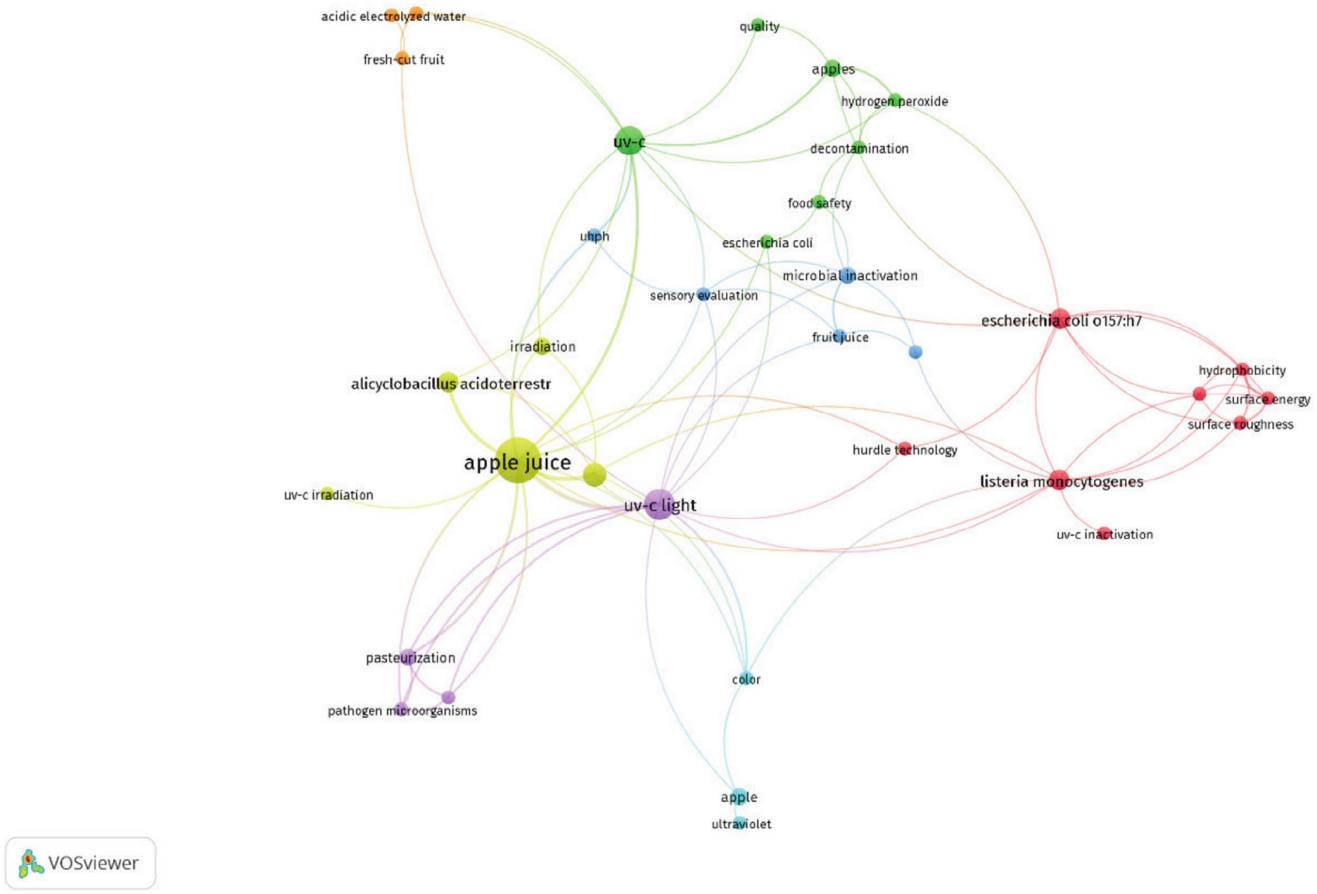
Co‐occurrence map of keywords generated with VOSviewer software.

The keywords highlight the main points of the article and the focus of research in this field. Figure 6 shows the co‐occurrence analysis of keywords, conducted with VOSviewer software. The network analysis identified seven thematic clusters, shown in different colors (red, green, purple, yellow, orange, light blue, and dark blue), which structure the research field on the application of UV‐C radiation in apples and apple products. VOSviewer analyzes how often terms appear together in the same articles. If they frequently co‐occur, the software considers them to have high semantic similarity, resulting in greater co‐occurrence strength. Each cluster represents a subset of terms with strong co‐occurrence.

The analysis included all keywords extracted from the 52 articles reviewed. The size of each node is proportional to the number of occurrences of the corresponding term, and the cluster colors indicate groups of words with stronger associations.

Cluster 1, shown in red, contains eight keywords and addresses the effects of UV‐C technology on food pathogens such as *E. coli* O157:H7 and *L. monocytogenes* , focusing on microbiological inactivation and emphasizing physicochemical properties with terms like surface roughness and hydrophobicity. Cluster 2, highlighted in green, includes seven keywords and centers on food safety and quality, associating terms such as food safety, decontamination, hydrogen peroxide, and quality, and characterizes research that combines UV‐C with oxidants for apple decontamination. Cluster 3, highlighted in dark blue and containing five keywords, focuses on microbiological efficiency and sensory impact studies. It brings together terms such as fruit juice, microbial inactivation, HHP, and sensory evaluation, reflecting the search for non‐thermal apple juice processes that are both microbiologically effective and maintain sensory acceptance by consumers. Cluster 4, in yellow, also contains five keywords and is notable for terms such as *Alicyclobacillus acidoterrestris* and *P. expansum* , emphasizing the role of UV‐C irradiation in the microbiological stability of apple juice, which is the most frequently used keyword by authors within this research topic. Cluster 5, in lilac, combines UV‐C light with thermal pasteurization and includes four keywords. Cluster 6, in light blue, contains three keywords—apple, color, and ultraviolet—indicating concern with visual and oxidative changes in products subjected to UV‐C treatment. Finally, Cluster 7, in orange, stands out by grouping three keywords and represents the emerging area of combined technologies, addressing acidic and neutral electrolyzed water in fresh‐cut fruit as alternatives to conventional sanitizers in minimally processed products.

### 3.2 Shortwave Ultraviolet Radiation (UV‐C) and Its Principle of Action

Ultraviolet light is a region of the electromagnetic spectrum that includes wavelengths between 100 and 400 nm and is traditionally divided into three main bands: UV‐A (315 to 400 nm), UV‐B (280 to 315 nm), and UV‐C (100 to 280 nm) (Nicolau‐Lapeña et al. 2023, 2022; Rios de Souza et al. 2020; Menezes et al. 2019).

UV‐C radiation is considered the most germicidal of the three bands because microbial DNA absorbs UV photons most efficiently in this range, with peak absorption at approximately 260 nm. Radiation at 253.7 nm, typically emitted by low‐pressure mercury lamps (LPM), is especially effective because it closely matches this absorption maximum. These lamps emit about 85% of their energy at this wavelength, contributing to their high efficiency in microbial inactivation (Akwu 2023; Green et al. 2020; Menezes et al. 2020).

The primary damage caused by UV‐C light is the formation of pyrimidine dimers. Many microorganisms can repair this damage through two mechanisms: (i) photoreactivation, which is light‐dependent and requires specific wavelengths between 300 and 500 nm to complete the reactivation process, and (ii) nucleotide excision (dark repair), which is light‐independent and involves multiple steps to reverse the formation of these dimers and the damage induced by UV‐C (Akgün and Ünlütürk 2017; Yin et al. 2015).

Recognized as a non‐thermal processing technology (Gabriel 2015; Gayán et al. 2015), UV‐C has emerged as a promising alternative to thermal treatments such as pasteurization for food preservation in the food industry. The US Food and Drug Administration (FDA) has approved its use at a wavelength of 254 nm as an alternative to thermal pasteurization for reducing human pathogens in fruit juices (Casco et al. 2025; Islam, Patras, Pokharel, et al. 2016; Islam, Patras, Pokharel, et al. 2016; Gouma et al. 2015a, 2015b). Its primary objective is the inactivation of microorganisms, and it is capable of achieving a 99.999% reduction in pathogen concentration (equivalent to a 5 log reduction) (Dias 2017; Islam, Patras, Pokharel, et al. 2016).

Although the 254 nm wavelength is the most common and traditionally studied, new sources such as UV‐C radiation‐emitting diodes (UV‐LEDs) are listed in Table 1. At wavelengths near 277 nm (266–280 nm), UV‐LED light has been associated with more effective inactivation of food pathogens compared to 253.7 nm in some studies. This increased efficacy may result from greater overlap of the emission spectrum with the germicidal action spectrum of the organism, additional inactivation mechanisms, or operational advantages such as the absence of mercury and smaller size (Corson et al. 2025; Green et al. 2020; Rios de Souza et al. 2020; Xiang et al. 2020).

**TABLE 1:** Comparison of LED sources with mercury lamps.

Wavelength (nm)	Typical source	Performance (compared to 254 nm)	References
222	LEDs (Far UV)/Excimer lamps (KrCl*)	In apple juice, it showed greater inactivation of *E. coli* O157:H7 than at 254 and 282 nm at similar fluences	Yin et al. (2015)
277	LEDs	The inactivation rate for *P. expansum* spores in saline solution and on the surface of apples was consequential higher than at 253.7 nm	Rios de Souza et al. (2020)
279	LEDs	It requires relevantly lower doses than 254 nm to inactivate Hepatitis A virus (HAV) in PBS and coconut water, possibly due to faster damage to viral proteins	Corson et al. (2025)
282	LEDs (Far UV+)	Apple juice showed greater penetration depth (0.074 cm) than at 222 nm or 254 nm	Orlowska et al. (2015)

The technological shift from low‐pressure mercury lamps (LPM) to UV‐C LEDs in the food industry requires a detailed analysis of operating costs, energy efficiency, and compliance with international regulatory standards. While mercury lamps have traditionally served as the primary source of UV‐C radiation for disinfection, ultraviolet light‐emitting diodes (UV‐LEDs) have emerged as a promising alternative, offering long‐term economic benefits despite being a newer technology (Rios de Souza et al. 2020; Akgün and Ünlütürk 2017; Orlowska et al. 2015). A key advantage of UV‐LEDs is that they do not require a warm‐up period and reach maximum irradiance immediately, unlike low‐pressure mercury lamps, which can take up to 30 min to stabilize.

In addition, mercury lamps have a relatively short lifespan, ranging from 4000 to 10,000 h, and are structurally fragile because they are made of glass, which poses a potential risk of physical and chemical contamination in food processing environments (Green et al. 2020; Rios de Souza et al. 2020; Akgün and Ünlütürk 2017). In contrast, UV‐LEDs can operate for more than 100,000 h and are more robust and impact‐resistant, enabling the development of more compact and flexible reactors (Xiang et al. 2020; Rios de Souza et al. 2020; Akgün and Ünlütürk 2017). Another important consideration is the presence of mercury in conventional light bulbs, which entails additional costs for proper disposal and potential risks to human health and the environment (Green et al. 2020; Akgün and Ünlütürk 2017). UV‐LEDs, on the other hand, are mercury‐free, align more closely with sustainability guidelines, and contribute to reducing environmental liabilities (Corson et al. 2025; Kim et al. 2021; Rios de Souza et al. 2020).

In addition, regulatory and environmental factors have driven the development of new UV emission technologies. The Minamata Convention on Mercury (2020) establishes global guidelines for reducing the use of mercury, encouraging the replacement of low‐pressure mercury lamps (LPM) with alternative technologies such as UV‐LEDs (Corson et al. 2025). Regulatory agencies, including the FDA and Health Canada, have required robust data on the effective radiation dose and microbiological efficacy of new wavelengths emitted by LEDs, such as 263 and 279 nm, before authorizing their industrial‐scale application. This underscores the importance of developing and validating safer, more efficient, and environmentally sustainable technologies for juice processing (Corson et al. 2025; Akwu 2023; Orlowska et al. 2015).

Limitations of the technology include low light penetration in cloudy products or those with suspended solids (turbidity), as well as the shadowing effect, which can prevent radiation from fully reaching microorganisms (Sauceda‐Gálvez et al. 2021; Graça et al. 2020; Gayán et al. 2015). Shading occurs mainly on the surface of apples due to the presence of microcracks, pores, depressions, and other topographical irregularities in the skin (Green et al. 2020; Rios de Souza et al. 2020; Adhikari et al. 2015). In these structures, microorganisms can remain lodged and physically protected from direct UV‐C radiation, which hinders their inactivation and can result in the so‐called “tail” effect in inactivation curves, characterized by a plateau in microbial reduction even with an increase in the applied dose (Nicolau‐Lapeña et al. 2022; Green et al. 2020).

Turbidity, however, is the main limitation in juice processing, caused by suspended solids, fibers, and macromolecules (Casco et al. 2025; Menezes et al. 2019; Gouma et al. 2015a). These particles absorb UV‐C radiation and cause light scattering, reducing penetration and making it difficult for the radiation to reach microorganisms within the liquid (Nicolau‐Lapeña et al. 2022; Gok 2021; Sauceda‐Gálvez et al. 2021). Additionally, the effectiveness of the technology depends on the type of fruit and the topography of its surface, as microorganisms may be concealed in fruits with rough or irregular surfaces, resulting in lower reduction effects (Nicolau‐Lapeña et al. 2023; Kim et al. 2021; Adhikari et al. 2015).

#### 3.2.1 Use of UV‐C in the Sanitization of Food and Other Products

UV‐C is considered an advantageous technology because it inactivates a wide range of pathogenic and spoilage microorganisms with minimal changes to the nutritional and sensory quality of food (Casco et al. 2025; Gouma et al. 2015b; Gayán et al. 2015). However, the effectiveness of this technology is strongly influenced by the morphological characteristics of the fruit surface (Adhikari et al. 2015; Syamaladevi et al. 2015). Smooth, less hydrophobic surfaces, such as those of apples and pears, result in higher inactivation rates, while rougher, more uneven surfaces, such as those of melons, strawberries, and raspberries, or those with cracks, cavities, or trichomes, can cause a shading effect that protects microorganisms from UV‐C light and requires higher doses for inactivation (Nicolau‐Lapeña et al. 2023, 2022; Rios de Souza et al. 2020; Adhikari et al. 2015).

In fruit juices, the use of UV‐C radiation has become popular during processing (Gabriel and Ancog 2019). However, the low penetration depth of UV‐C light is the main obstacle to its application in liquids (Tirono 2023; Yin et al. 2015). Its effectiveness is limited by the turbidity of the medium, which results from high UV absorption by components such as suspended solids, insoluble fibers, pigments, sugars, and organic acids. Strategies such as thin‐film treatment are used to maximize surface area and decrease product depth (Orlowska et al. 2015; Gok 2021; Nicolau‐Lapeña et al. 2022). In addition to its use in liquid foods, UV‐C is widely used for water disinfection, including wastewater and hydroponic nutrient solutions (Casco et al. 2025; Tremarin et al. 2017a). UV‐C is also applied in the decontamination of egg and shellfish surfaces and for the microbiological decontamination of surfaces in general (Sauceda‐Gálvez et al. 2021, 2020).

The UV‐C radiation fluence ranges vary significantly depending on the research objective (inactivation of pathogens or fungi, or degradation of mycotoxins) and the food matrix used (fruit surface or juices) (Table 2).

**TABLE 2:** Comparison of target microorganisms and the fluence range applied.

Target microorganism	Fluence range/Applied doses	References
*E. coli* O157:H7 and *L. monocytogenes* on fruit surfaces	0 a 10.5 kJ/m^2^	Adhikari et al. (2015)
*E. coli* (pathogenic and surrogate strains) in apple juice	0 to 514.32 mJ/cm^2^ (at 254 nm)	Orlowska et al. (2015)
Polyphenols, vitamins, and microorganisms in apple juice	0 to 240 mJ/cm^2^	Islam, Patras, Pokharel, et al. (2016), Islam, Patras, Pokharel, et al. (2016)
Whole ‘Gala’ apples (quality and phenolics)	0 to 4.10 kJ/m^2^	Dias (2017)
Metabolic reactions in fresh‐cut apples	6, 12, and 24 kJ/m^2^	Hasan et al. (2017)
Ascospores of *N. fischeri* in apple juice	0 to 120 kJ/m^2^	Menezes et al. (2019)
Ascospores in clarified apple juice	7.2 to 21.5 J/mL	Sauceda‐Gálvez et al. (2019)
Yeasts in apple slices	2.5 to 10 kJ/m^2^	Graça et al. (2020)
*E. coli* and *L. monocytogenes* on apple peel and lettuce	0 to 1000 mJ/cm^2^	Green et al. (2020)
*E. coli* and *A. niger* in apples (Advanced oxidative process)	11.7 to 62.3 kJ/m^2^	Ho et al. (2020)
*A. fischeri* and *P. niveus* in apple juice	Up to 25 kJ/m^2^	Menezes et al. (2020)
Bacterial spores in cloudy apple juice	25 to 125 J/mL	Sauceda‐Gálvez et al. (2021)
*E. coli, Salmonella*, and *L. monocytogenes* in peel and juice	Peel: up to 10,665.9 mJ/cm^2^; juice: up to 3644.1 mJ/cm^2^	Nicolau‐Lapeña et al. (2022)
Microbial inactivation and vitamins in opaque juice	0 to 160 mJ/cm^2^	Akwu (2023)
Patulin in apples and juices	Surface: 8.8 to 35.1 kJ/m^2^; Juice: up to 450.6 kJ/m^2^	Nicolau‐Lapeña et al. (2023)
Combined strategy in apple juice	1271 mJ/cm^2^ (fixed)	Casco et al. (2025)
Hepatitis A virus in juice and coconut water	Up to ~5.14 mJ/cm^2^ (in juice)	Corson et al. (2025)

The feasibility of applying UV‐C radiation on an industrial scale has been widely discussed in the literature, with a primary focus on its microbiological efficacy, the challenges related to the optical properties of products, and its potential for low‐cost operation (Sauceda‐Gálvez et al. 2021; Gouma et al. 2015a, 2015b).

Regarding industrial feasibility and technical challenges, UV‐C is approved by the FDA for juice pasteurization, requiring a 5‐log reduction in pathogens. However, its implementation faces physical obstacles such as limited penetration depth in turbid liquids or those with a high absorption coefficient (Gok 2021; Islam, Patras, Pokharel, et al. 2016; Gouma 2015). For apple juice, 90% of radiation absorption occurs within the first 0.67 mm (Nicolau‐Lapeña et al. 2023). To address this, the industry uses thin‐film reactors, where juice passes through thin layers, or systems that induce turbulence (Dean vortex technology), ensuring all components of the liquid are exposed to the lamp (Gok 2021; Gouma et al. 2015a). In terms of industrial feasibility, the use of moderate heat (45°C–60°C), ultrasound, or additives (such as DMDC or natamycin) is also considered (Casco et al. 2025; Gabriel 2015). Through this synergy of technologies, the required safety standards are met with lower doses and shorter processing times, thereby preventing sensory damage to the product (Gouma et al. 2015a, 2015b). When analyzing energy consumption, energy efficiency is recognized as a competitive advantage of UV‐C radiation. Recent studies report that the energy consumption of this technology may be only 6% to 8% of that of conventional treatments such as heat, ohmic, microwave, or high‐pressure methods (Nicolau‐Lapeña et al. 2023). The transition to UV‐LEDs offers even greater viability, as they are more energy‐efficient, mercury‐free, capable of instant on/off switching, and can operate at ambient temperatures, reducing operating and maintenance costs (Rios de Souza et al. 2020; Xiang et al. 2020). In addition to economic and market advantages, along with low energy consumption, the industrial viability of UV‐C is supported by its low initial investment compared to technologies such as High Hydrostatic Pressure (HHP); UV‐C requires a smaller investment, making it accessible to small‐scale processors (Nicolau‐Lapeña et al. 2022). Furthermore, liquids treated with UV‐C better retain their flavor, color, and vitamin content (such as vitamin C) than thermally pasteurized juices, thereby increasing their added value (Yang et al. 2019).

#### 3.2.2 Legislation and Regulation of UV‐C Technology Use

Current regulations on the use of UV‐C address the approval of this technology in the United States (FDA) and the European Union. In response to outbreaks of juice‐related illnesses, the US Food and Drug Administration (US FDA) ratified the Juice Hazard Analysis Critical Control Point (Juice HACCP) and established a requirement for logarithmic reduction of resistant microorganisms, mandating that juices undergo processing steps capable of reducing the population of a target pathogen by at least 5 logarithmic cycles (Gayán et al. 2015; Gouma et al. 2015a, 2015b; Gabriel and Ancog 2019; Gabriel 2015).

The US FDA has approved the use of UV‐C light, specifically at a wavelength of 254 nm, for microbial reduction in food and juices (Corson et al. 2025; Chen et al. 2016; Adhikari et al. 2015; Yin et al. 2015). The regulation governing its application, 21 CFR Part 179.39, refers to “ultraviolet radiation for food processing and treatment” (Akwu 2023; Nicolau‐Lapeña et al. 2022; Gómez et al. 2015; Syamaladevi et al. 2015).

Since 1999, the European Union has adopted Directive 1999/2/EC, which harmonizes legislation among its member countries regarding the treatment of food and food ingredients using only ionizing radiation. The directive aims to reduce the risk of disease and food spoilage caused by pathogenic microorganisms or contaminants, decrease food losses due to physiological changes, and maintain the health of plant materials. The European Community defines sources of ionizing radiation as those that emit gamma rays (60Co and 137Cs), X‐rays, and electrons, applied to food at an average overall dose of < 10 kGy (European Parliament and Council of the European Union 1999).

Under current legislation, the use of non‐ionizing radiation, such as UV‐C light, is not explicitly addressed in European laws targeting the reduction of contaminating microorganisms in food. However, EU Implementing Regulation 2017/2470 restricts the use of UV‐C radiation to increasing vitamin D in mushrooms (*Agaricus bisporus*), bread, bread yeast (*Saccharomyces cerevisiae*), and milk, designating these as novel foods after the application of this technology (Nicolau‐Lapeña et al. 2022).

This regulatory context highlights a significant gap in European regulations concerning the use of non‐ionizing UV‐C radiation for food decontamination. Unlike the United States, which recognizes UV‐C as a non‐thermal processing alternative, European legislation has not yet established specific guidelines for food safety applications (Tchonkouang et al. 2023). This more cautious approach is partly due to the regulatory philosophy of the European Union, which emphasizes risk assessment and precaution when evaluating new food processing technologies (Smedt and Vos 2022). Consequently, UV‐C radiation treatments require case‐by‐case approval, particularly when they may affect food composition or generate chemical by‐products (Smedt and Vos 2022).

Internationally, different regulatory frameworks can affect the acquisition and commercialization of UV‐C radiation. Harmonizing food safety guidelines plays an important role in facilitating foreign trade and ensuring consistent consumer protection across all regions (Keener and Koutchma 2022). In this context, the Codex Alimentarius, established by the Food and Agriculture Organization of the United Nations and the World Health Organization, provides a set of internationally recognized standards for food safety and processing practices (Fortin 2023). Although the Codex Alimentarius focuses on hazard control, hygiene, and risk analysis, it contributes to global regulatory alignment and can support future discussions on the safe implementation of emerging food processing technologies, such as UV‐C radiation (Fortin 2023).

#### 3.2.3 UV‐C Radiation on Apples and By‐Products

##### 3.2.3.1 UV‐C and Fresh Apples

The main microbial agents responsible for post‐harvest deterioration of fresh apples are *P. expansum* and *Botrytis cinerea*, both known for their considerable impact on the storage and commercial quality of the fruit. *P. expansum* causes blue mold, resulting in severe tissue degradation, accelerated senescence, and loss of firmness. In addition to physical and economic losses, the production of the mycotoxin patulin poses a major food safety risk and has been associated with contamination incidents in various production chains worldwide (Rios de Souza et al. 2020; Nicolau‐Lapeña et al. 2023).

*B. cinerea* , which causes gray mold, is more common during prolonged storage and contributes to secondary rot. The main methods for controlling these microorganisms include non‐thermal technologies and chemical control with sanitizers and fungicides (Luciano‐Rosario et al. 2025; Adhikari et al. 2015; Konstantinou et al. 2011).

Sodium hypochlorite is widely recognized and commonly used as an aqueous sanitizer for disinfecting fresh produce, making it the most frequent method for industrial‐scale washing. Its application often involves chlorinating water in discharge tanks, a practice documented in the apple processing industry (Green et al. 2020; Adhikari et al. 2015). Hypochlorite disrupts microbial cytoplasmic membranes, irreversibly inhibits enzymes, and denatures proteins. However, its effectiveness is limited against high microbial loads, and its use generates large volumes of wastewater that require treatment (Luciano‐Rosario et al. 2025).

To develop alternatives to chlorine‐based sanitizers, some studies have investigated emerging methods for sanitizing apples using UV‐C light from different sources with varying fluence levels and exposure times (Phonyiam et al. 2021; Dias 2017; Adhikari et al. 2015; Pataro et al. 2015).

The primary mechanism of microbial inactivation by UV‐C on apple peel is direct photochemical damage to DNA, resulting in the formation of pyrimidine dimers that prevent cellular replication (Sauceda‐Gálvez et al. 2021, 2019; Gabriel and Ancog 2019). The efficacy of this technology depends greatly on surface morphology; because apples have relatively smooth and less hydrophobic skins, they allow more direct interaction between the radiation and microorganisms (Nicolau‐Lapeña et al. 2022; Corrêa 2020). In whole fruits, low doses of UV‐C can induce a biological response (hormesis), stimulating the biosynthesis of secondary metabolites and defense enzymes that aid in postharvest preservation (Dias 2017; Tremarin et al. 2017b; Pataro et al. 2015).

Phonyiam et al. (2021) tested the antimicrobial effect of UV‐C light on apples artificially inoculated with *Colletotrichum gloeosporioides*, which causes post‐harvest rot. The experiment included three batches of apples (*n* = 60): the first batch was exposed to a fluence of 3 kJ/m^2^ at a distance of 25 cm from the UV‐C source before inoculation, the second batch was exposed to the same radiation parameters after inoculation, and the third batch was not irradiated (control). All batches were stored for 16 days at 25°C. Exposure to UV‐C radiation significantly reduced the diameter of lesions caused by *C. gloeosporioides* (control: 5.04 ± 0.08 cm; pre‐inoculation: 4.51 ± 0.15 cm; post‐inoculation: 4.49 ± 0.11 cm), regardless of exposure timing, compared to the control group. However, UV‐C treatment before inoculation was more effective in suppressing this microorganism.

Adhikari et al. (2015) observed marked reductions in surface contamination of apples by *E. coli* O157:H7 (2.9 log CFU/g) and *L. monocytogenes* (1.6 log CFU/g) after applying 0.92 kJ/m^2^ of UV‐C, supporting the potential of this technology for pathogen control.

In addition to its antimicrobial effect, Dias (2017) observed improvements in antioxidant activity and phenolic compound content, as well as maintenance of firmness, pH, soluble solids content (SST), titratable acidity (ATT), and the SST/ATT ratio, concluding that UV‐C had an additional effect on maintaining the physicochemical quality of the fruit. Pataro et al. (2015) applied UV‐C light at a distance of 30 cm from the source with fluences of 2 J/cm^2^ (1 h of exposure) and 4 J/cm^2^ (2 h of exposure) on both sides of the fruit, using pulsed light. After 28 days of storage, they observed no notable changes in the color of irradiated fruits, but increases in soluble solids, antioxidant activity, and total phenolic compounds, indicating positive effects on nutritional composition.

Patulin, a secondary fungal metabolite classified as a mycotoxin, is produced mainly by fungi such as *P. expansum* , as well as *Aspergillus* and *Byssochlamys* species. It can cause acute and subacute toxicity, as well as chronic symptoms including immunotoxicity, hepatotoxicity, and gastrointestinal and neurological problems (Rios de Souza et al. 2020). Due to its potentially harmful effects and resistance to most physical and chemical treatments, the presence of patulin in food is legally regulated, with the maximum limit set by the US Food and Drug Administration (FDA) and the China Food and Drug Administration at 50 μg/kg for fruit juices (Nicolau‐Lapeña et al. 2023).

Nicolau‐Lapeña et al. (2023) demonstrated that applying two separate fluences (UV1: 8.8 kJ/m^2^ and UV2: 35.1 kJ/m^2^) effectively reduced patulin, a highly relevant mycotoxin associated with outbreaks from contamination of apples and their by‐products produced by *P. expansum* (CMP‐1). Reductions of 2.7 log CFU/g (UV1) and 99.9% of the mycotoxin (UV2) highlight the importance of this technology in addressing toxigenic risks.

Excessive irradiation can have harmful effects on both the physical structure and chemical composition of food. It can rupture the cell membranes of apples, promoting progressive dehydration and increasing mass loss, which leads to the loss of functional compartmentalization, greater contact between enzymes and substrates, and accelerated oxidation reactions (Chen et al. 2016; Gómez et al. 2015). At very high intensities (e.g., 100–125 mW/cm^2^), the thermal effect accompanying radiation can degrade natural substances, resulting in immediate browning (Tirono 2023).

Overall, the results indicate that UV‐C radiation applied to fresh apples can reduce spoilage and pathogenic microorganisms while maintaining microbiological stability during storage. This is an advantage over chlorine, whose effectiveness is often reduced by the presence of organic matter (Ho et al. 2020; Rios de Souza et al. 2020).

##### 3.2.3.2 UV‐C × Minimally Processed Apple (Freshcut)

The production of minimally processed foods (MPF) is driven by consumer demand for products with fresh characteristics and higher nutrient retention, in contrast to traditional thermal pasteurization, which can degrade sensory and nutritional properties (Akwu 2023; Tirono 2023; Yin et al. 2015; Nicolau‐Lapeña et al. 2022). The main challenge during processing and storage of these products is to ensure microbiological safety and prevent enzymatic browning caused by the cutting steps in minimal processing (Corrêa 2020).

Studies on minimally processed apples treated with UV‐C radiation have shown significant effects on physicochemical quality and microbiological reduction (Chen et al. 2016). Kim et al. (2021) found that a combined treatment (UV‐C plus slightly acidic electrolyzed water (SAEW) and fumaric acid (FA) for 3 min at 10°C and 15°C for 7 days) improved the color parameters “L” and “a” of the fruits. From a microbiological perspective, this treatment was also effective against strains of *L. monocytogenes* inoculated on the surface of the samples, resulting in a greater disinfection effect (reduction of 0.51 to 2.63 log CFU/g) than treatment with SAEW alone or the partial combined treatment (SAEW plus FA), which achieved reductions between 0.31 and 2.17 log CFU/g. The authors chose a barrier technology (UV‐C plus slightly acidic electrolyzed water and fumaric acid) because previous studies (Guo et al. 2021; Hussain et al. 2019; Ngnitcho et al. 2017) reported a synergistic effect for microbial reduction superior to treatment with electrolyzed water alone.

Chen et al. (2017) used a combined treatment of UV‐C and electrolyzed water at 10°C for 14 days and demonstrated that, during storage of minimally processed apples, *Salmonella enteritidis* remained stable in treated samples, while control samples showed an increase of up to 5 log CFU/g. Regarding physicochemical quality, this combined treatment did not compromise the color, firmness, or flavor of the fresh‐cut apples, although it caused a slight deterioration in the sensory attributes of flavor (Chen et al. 2017).

Graça et al. (2020), using the same combined treatment of UV‐C and electrolyzed water at 4°C for 9 days, found that despite reductions in yeasts such as *Candida sake* , *Hanseniaspora uvarum* , *Pichia fermentans* , and *Metschnikowia pulcherrima* (10^7^ CFU/mL), the shelf life of the samples was not extended. This indicates limitations of the method for UV‐C and electrolyzed water treatment of minimally processed apples.

Electrolyzed water, often used with UV‐C, is an environmentally friendly disinfectant produced by the electrolysis of saline solutions, such as sodium chloride, that pass through an electrolytic cell. This process generates a mixture of potent antimicrobial substances. Although its mechanism of action is not yet fully understood, it is known to be related to its oxidative potential (Graça et al. 2020). The biocidal activity of UV‐C and electrolyzed water occurs through the oxidation of cellular components, including proteins, lipids, and nucleic acids, by disrupting the cell wall and membrane, which leads to the loss of intracellular constituents (Kim et al. 2021).

Green et al. (2020) also reported satisfactory reductions of *L. monocytogenes* and *E. coli* O157:H7 in minimally processed apples treated with UV‐C radiation. Two different UV‐C sources were used: (i) mercury lamps (fluence of 8.55 mW/cm^2^ at 10 cm from the emitting source) and (ii) LEDs (fluences of 2.33 and 2.41 mW/cm^2^ at 25°C and 4°C, respectively, both at 14.4 cm from the emitting source), reinforcing the effectiveness of this technology against pathogenic microorganisms regardless of the UV‐C light source.

Enzymatic browning is a critical factor in minimally processed foods, as it directly affects their visual, sensory, and commercial quality. This process involves the natural degradation of processed foods by enzymes such as polyphenol oxidase which, when in contact with phenolic compounds and exposed to oxygen, accelerates browning as the enzyme and substrate interact after cutting (Yuan et al. 2022). According to these authors, applying UV‐C before and after processing for 5 min contributed positively to the visual and functional quality of minimally processed apples. In this study, after 14 days of storage at 4°C, the irradiated samples showed significantly lower enzymatic browning rates and improved antioxidant systems compared to the control samples.

##### 3.2.3.3 UV‐C × Apple Juice

The application of UV‐C radiation to apple juice has proven to be a promising alternative for microbiological control and quality preservation (Casco et al. 2025). The uniform distribution of fluence in juices is one of the main technical challenges in applying UV‐C radiation, due to the optical properties of the matrix. Apple juice, for example, is considered a highly opaque medium for UV‐C radiation, with transmission rates that can be < 0.01%. Compounds present in the juice, such as vitamin C and polyphenols, strongly absorb photons, limiting light penetration to very superficial layers, often on the order of millimeters—approximately 0.67 mm to achieve 90% absorption (Nicolau‐Lapeña et al. 2023, 2022; Gabriel 2015; Orlowska et al. 2015). Given this limitation, it is essential to use systems that promote greater fluid homogenization, such as turbulent flow regimes or the generation of Dean vortices, which facilitate the movement of particles from less exposed regions to areas near the radiation source, thereby increasing the likelihood that the entire microbial load is effectively exposed to the fluence required for inactivation (Gok 2021; Gabriel 2015).

Gabriel (2015) combined UV‐C radiation (15 W at a distance of 36 cm from the source) with ultrasound (frequencies alternating between 28, 45, and 100 kHz at 1 ms intervals) and observed a shorter time interval (51.5 min: US + UV‐C) for a 5 log CFU/mL reduction of *E. coli* O157:H7 isolates in apple juice samples compared to ultrasound alone (77.5 min: US), demonstrating a synergistic effect between the technologies.

Consistent results for the reduction of microorganisms in juices after UV‐C application were also reported by Tremarin et al. (2017a) when evaluating juice samples inoculated with *A. acidoterrestris* spores. Using fluences of 11.50 and 13.44 W/m^2^ for 8 min, reductions of up to 5 logarithmic cycles were observed in the juice samples, while a lower fluence (0.34 W/m^2^) achieved a similar effect after 25 min of exposure. This indicates that the efficiency of the treatment depends on the intensity and duration of radiation exposure. In addition to satisfactory microbiological effects (reductions of up to 99% in bacteria and 90% in fungi and yeasts) and an increase in product shelf life of at least three weeks, Yang et al. (2019) also reported positive impacts on quality, identifying increased soluble solids and acidity levels in apple juices subjected to UV‐C and subsequently stored at 4°C for 35 days. Corroborating these findings, Pérez‐Lorenzo et al. (2022) reported significant reductions of lactic acid bacteria and acetic acid bacteria in natural apple cider subjected to UV‐C irradiation, reinforcing the potential of the technology for fermented apple‐derived beverages.

The debate between continuous and batch processing systems for UV‐C radiation treatment is central to transitioning from laboratory research to industrial viability, especially for fruit juices and the surfaces of fresh produce.

Continuous systems are designed to process large volumes and form the basis of the commercial viability of UV‐C technology. Their reactors can be thin‐film types—sequential annular reactors where the fluid passes through a very narrow slit (2 to 3 mm) to shorten the distance the light must travel—or Dean Vortex‐type reactors, which use the helical geometry of the tube to create secondary vortices that promote optimal fluid mixing, ensuring that even particles in opaque fluids are drawn close to the light source (Nicolau‐Lapeña et al. 2022; Gok 2021; Sauceda‐Gálvez et al. 2021, 2020; Gouma et al. 2015a, 2015b).

It is estimated that energy consumption in continuous UV‐C systems accounts for only 39% of the energy used in conventional thermal pasteurization, and this treatment model is ideal for ensuring that all parts of the product receive an equivalent dose of radiation (Sauceda‐Gálvez et al. 2021).

Batch systems are frequently used in laboratory studies to establish fundamental inactivation parameters. Their key features and advantages include: (i) dosing accuracy, where equipment such as the collimated beam delivers highly precise UV‐C doses by ensuring the sample is well mixed and factors such as divergence and reflection are quantified; and (ii) experimental control, which facilitates the study of inactivation kinetics of specific microorganisms and the degradation of toxins under controlled conditions (Akwu 2023; Nicolau‐Lapeña et al. 2023; Hasan et al. 2017; Islam, Patras, Pokharel, et al. 2016).

Overall, available studies show that UV‐C radiation, used alone or combined with other technologies, has significant potential for inactivating microorganisms and maintaining apple juice quality. However, its effectiveness depends on applied fluence, exposure time, storage conditions, and juice characteristics such as turbidity and opacity, which can reduce radiation efficiency and require fluence adjustments or complementary strategies (Sauceda‐Gálvez et al. 2021; Gayán et al. 2015; Gouma et al. 2015a). Therefore, research on combining UV‐C with other emerging approaches is especially relevant, a topic discussed in the next section.

### 3.3 UV‐C and Over‐Irradiation

Excessive irradiation can have harmful effects on both the physical structure and chemical composition of food; it can rupture cell membranes, promote progressive dehydration, and exacerbate mass loss, resulting in the loss of functional compartmentalization, increased contact between enzymes and substrates, and accelerated oxidation reactions (Chen et al. 2016; Gómez et al. 2015). At very high intensities (e.g., 100–125 mW/cm^2^), the thermal effect accompanying radiation can degrade natural substances, resulting in immediate browning (Tirono 2023).

The sensory effects of UV‐C irradiation depend on the food matrix and the applied dose. For color and appearance, the main sign of overexposure in minimally processed apples is increased surface browning. Studies indicate that low or moderate doses, such as 3 kJ/m^2^, may not cause immediate color changes; however, higher doses can lead to noticeable browning during storage (Gómez et al. 2015; Syamaladevi et al. 2015). For apple juice, applying excessive radiation doses, especially those above 100 J/mL in recirculation systems, is linked to increased browning and turbidity (Sauceda‐Gálvez et al. 2021).

On the other hand, regarding product firmness, studies indicate that this parameter is generally not negatively affected by UV‐C radiation compared to the control treatment, representing an advantage over conventional thermal processes, which can cause greater textural degradation (Shin et al. 2024; Dias 2017; Chen et al. 2016).

Gok (2021) state that juices treated with high doses of UV‐C showed no important changes in appearance, color, odor, and flavor scores among trained sensory panels, indicating that these attributes were preserved.

### 3.4 UV‐C Associated With Other Technologies

**FIGURE 7:**
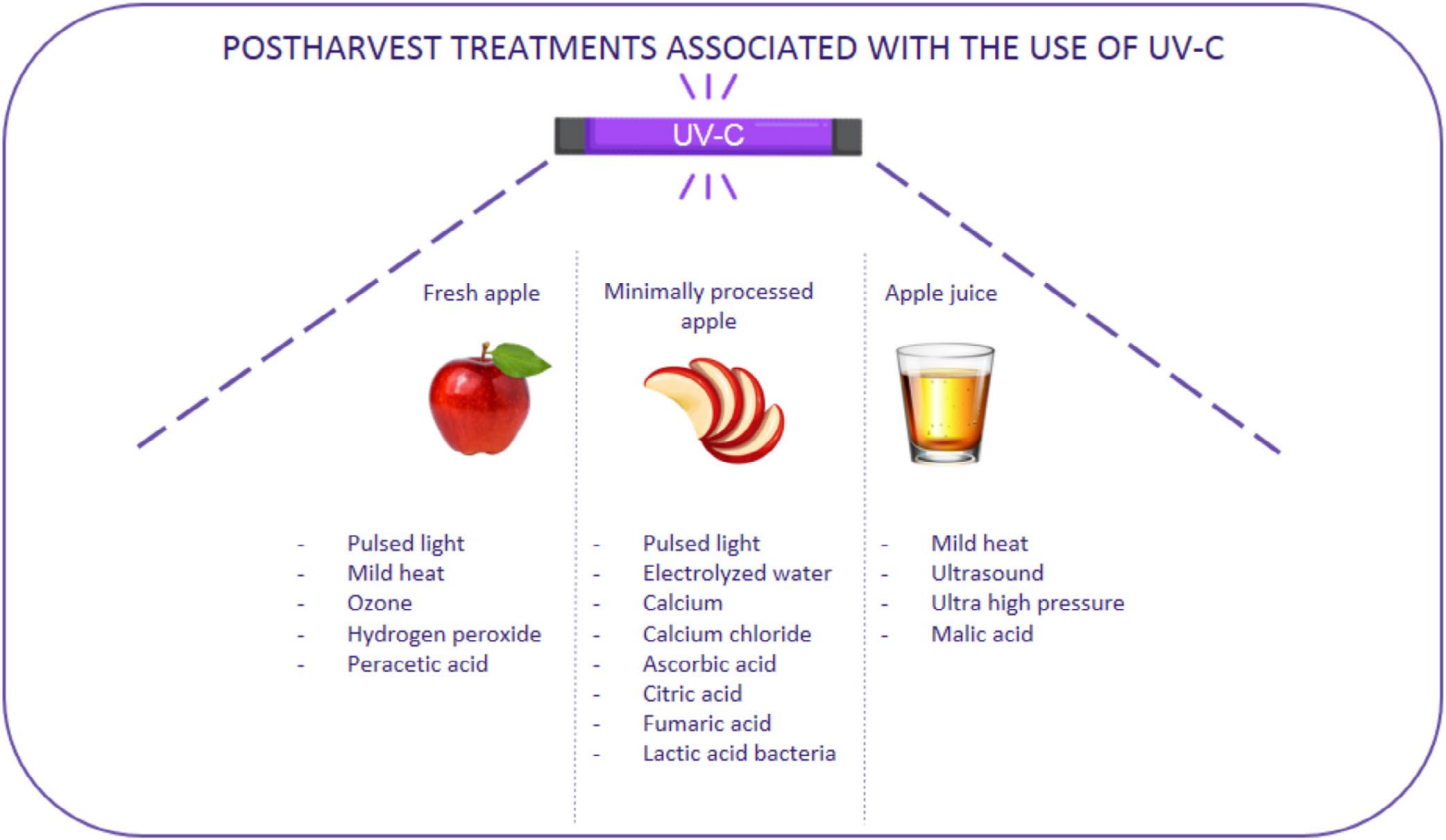
Combining UV‐C with other technologies in fresh apples, minimally processed apples, and apple juice.

The combination of UV‐C radiation with other emerging technologies (hurdle technology) has been studied as an alternative to enhance microbiological effects while preserving the sensory and nutritional attributes of apple products (Figure 7 and Table 3). This approach demonstrates that combining methods often produces a synergistic or additive effect that is superior to individual treatments for both food safety and quality (Casco et al. 2025; Kim et al. 2021).

**TABLE 3:** Combining UV‐C with other technologies in fresh apples, minimally processed apples, and apple juice.

Product	Association	Main results	References
Fresh apple	UV‐C + Pulsed light	Increased antioxidants; maintained quality	Pataro et al. (2015)
	UV‐C + Ozone	Reduced *L. monocytogenes*	Murray et al. (2018)
	UV‐C + + Heat	Degraded insecticides	Ho et al. (2020)
	UV‐C + Peracetic acid	Pathogen inactivation ( *E. coli* and *L. monocytogenes* ); firmness maintained	Shin et al. (2024)
	UV‐C + Calcium + Ascorbic acid + Calcium chloride	Microbial reduction (mesophiles, fungi, and yeasts) caused darkening	Gómez et al. (2015)
	UV‐C + Citric acid	Reduced browning; maintained texture	Chen et al. (2016)
	UV‐C + Electrolized water	Reduced *Cronobacter sakazakii*	Santo et al. (2016)
	UV‐C + *L. plantarum*	Reduced pathogens ( *S. enteritidis* ); maintained quality	Chen et al. (2017)
	UV‐C + Pulsed light	Altered flavor	Hasan et al. (2017)
	UV‐C + Electrolized water	Inactivated enzymes; reduced browning	Graça et al. (2020)
	UV‐C + EW + Fumaric acid	Reduced yeasts, *L. monocytogenes* , and *S. aureus*	Kim et al. (2021)
Apple juice	UV‐C + Heat	Significant *L*. *monocytogenes* reduction	Gayán et al. (2015)
	UV‐C + Malic acid + Heat	Synergistic inactivation of *L. monocytogenes*	Gabriel and Ancog (2019)
	UV‐C + Heat + DMDC	5‐log *E. coli* reduction; saved time	Gouma et al. (2015a)
	UV‐C + HHP	Reduced *A. acidoterrestris* spores; high nutrient retention	Sauceda‐Gálvez et al. (2020)
	UV‐C + Ultrasound	Synergistic *E. coli O157:H7* inactivation	Gabriel (2015)
	UV‐C + Heat (60°C)	Saved 89% of processing time	Gouma et al. (2015a)
	UV‐C + Mild Heat	5‐log *S. cerevisiae* reduction; saved time	Gouma et al. (2015a)
	UV‐C + HHP	Increased polyphenols; altered flavor	Sauceda‐Gálvez et al. (2021)
	UV‐C + Temp (40°C)	Enriched bioactive compounds	Yıkmış et al. (2021)
	UV‐C + Ultrasound	5‐log *A. acidoterrestris* reduction	Tremarin et al. (2017b)
	UV‐C + Heat + Natamycin	Complete inactivation; increased antioxidants	Casco et al. (2025)
	UV‐C + HHP	Reduced *Talaromyces macrosporus*; increased antioxidants	Sauceda‐Gálvez et al. (2019)

Studies reinforce the potential of technologies based on UV‐C radiation and advanced oxidation processes (AOP) as promising alternatives to conventional apple decontamination methods, highlighting their antimicrobial action and preservation of sensory quality (Shin et al. 2024; Ho et al. 2020; Murray et al. 2018; Pataro et al. 2015).

Pataro et al. (2015) demonstrated that post‐harvest application of UV‐C (2–4 J/cm^2^) and pulsed light to apples stimulated the synthesis of bioactive compounds, increasing phenolic content and antioxidant activity without altering the pH, titratable acidity, or color of the fruit during storage. These results indicate a hormetic effect of UV‐C radiation, in which moderate doses induce beneficial metabolic responses in fruits.

Pulsed light technology is a form of radiation that induces stress in plant tissues, stimulating the biosynthesis of secondary defense metabolites with antimicrobial and antioxidant activity. By activating these defenses, its combination with UV‐C is an effective method for prolonging product freshness and preserving or even enhancing the content and activity of antioxidant compounds, thereby extending shelf life and improving nutritional value (Pataro et al. 2015).

Studies show that the response of antioxidants varies critically among matrices, depending on the applied dose and storage time. In whole or minimally processed fruits, UV‐C radiation often acts as a stressor that stimulates antioxidant synthesis (hormetic effect), increasing total phenolic content and antioxidant activity for up to 90 days of refrigerated storage (Dias 2017). Another study on the Annurca variety showed that UV‐C increased phenolic compound levels in the peel, noting a localized effect, as the increase is generally restricted to the surface layer (peel), with little or no change detected in the pulp of fresh fruit (Pataro et al. 2015).

In apple juice, the application of UV‐C radiation generally promotes the degradation or retention of bioactive compounds. Several studies report significant reductions in key polyphenols, such as chlorogenic acid and epicatechin, especially at higher radiation doses (Akwu 2023; Islam, Patras, Pokharel, et al. 2016; Islam, Patras, Pokharel, et al. 2016). Similarly, the use of UVC‐LEDs has been associated with decreased antioxidant activity and total phenolic content, demonstrating a dose‐dependent response (Xiang et al. 2020).

However, when commercially relevant doses, such as approximately 40 mJ/cm^2^, are used, the total phenolic content tends to be well preserved, with no significant changes (Islam, Patras, Pokharel, et al. 2016, 2016). Furthermore, some authors have reported an increase in the antioxidant activity of juice after UV‐C treatment, attributing this effect to the possible release of phenolic compounds previously bound to cell walls or to the inactivation of the enzyme polyphenol oxidase (PPO), which could reduce the oxidation of these compounds (Casco et al. 2025; Gok 2021). In general, while the application of UV‐C radiation to the surface of apples tends to stimulate a biological increase in antioxidant compounds associated with the plant tissue's defense mechanisms, in apple juice the bioactive compounds already present in the matrix are predominantly maintained or chemically degraded.

Murray et al. (2018) and Ho et al. (2020) investigated the use of AOP technology, which combines hydrogen peroxide, ozone, and UV‐C radiation, for microbial inactivation and chemical residue degradation in apples. Murray et al. (2018) achieved reductions of up to 3 log of *L. monocytogenes* in apples intended for caramel apple production, both on the surface and inside the fruit, by applying ozone gas followed by a continuous oxidative process with UV‐C at 54 mJ/cm^2^ for 20 min. In a similar experiment, Ho et al. (2020) demonstrated a 59% degradation of the pesticide chlorpyrifos, along with a reduction of more than 6 log in *E. coli* O157:H7 and *Aspergillus niger*, highlighting the potential of AOP as a dual‐function approach for both microbiological and chemical control.

More recently, Shin et al. (2024) proposed combining peracetic acid (PAA) and UV‐C light (280 nm) to control pathogens in water and on the surface of apples. They achieved complete inactivation of *E. coli* O157:H7 and *L. monocytogenes* in just 5 min, without significantly affecting color or firmness. The study highlights the synergistic effect between PAA and UV‐C (at fluences of 0–4 mJ/cm^2^ and 0–15 mL/cm^2^) in generating reactive oxygen species, which intensify damage to membranes and microbial DNA.

In general, authors agree that using UV‐C radiation alone or combined with other systems (PL, ozone, H_2_O_2_, or PAA) effectively reduces microbial load and, in some cases, stimulates defense compounds in fruits without compromising quality. However, gaps remain in standardizing doses and evaluating long‐term effects on sensory attributes, bioactive compounds, and residual chemical safety. Additionally, industrial scalability and associated energy costs continue to challenge large‐scale adoption of these technologies.

The studies analyzed assess the effectiveness of UV‐C light combined with chemical agents (calcium salts, ascorbic acid, citric acid, and electrolyzed water) and thermal treatments (blanching) to extend the shelf life of minimally processed apples. The primary focus is on microbial inactivation (native flora, yeasts, and pathogens such as *L. monocytogenes* , *S. aureus* , and *Cronobacter sakazakii* ) and the maintenance of quality attributes (color/browning, firmness, and viscoelastic properties) (Kim et al. 2021; Chen et al. 2016; Santo et al. 2016; Gómez et al. 2015).

The use of UV‐C light is approved by the Food and Drug Administration for decontaminating food and surfaces, and studies aim to optimize doses (fluences) and combinations to maximize microbial reduction while minimizing or avoiding negative effects on sensory and structural characteristics (Chen et al. 2016; Gómez et al. 2015).

There is consensus that UV‐C radiation is a valuable tool for reducing the initial microbial load and, consequently, decreasing the growth of microorganisms during refrigerated storage (Table 4). Gómez et al. (2015) reported that UV‐C light reduced the microbial load in calcium‐fortified apples (1.3 log CFU/g) and decreased growth during 7 days of cold storage (0.7 to 2.6 log CFU/g). Chen et al. (2016) and Kim et al. (2021), when combining UV‐C with other technologies (citric acid and electrolyzed water plus fumaric acid, respectively), observed reduced microbial growth and maintained bacterial counts at lower levels than the controls during storage.

**TABLE 4:** Microbial log reduction: UV‐C versus combined UV‐C.

Food matrix	Target microorganism	Single UV‐C reduction (log)/Reference	Combined UV‐C reduction (log)/Reference	Combined technology
Clarified apple juice	*E. coli* /Pathogens	1.2–4.6/Akwu (2023), Gouma et al. (2015b)	> 6.0/Gouma et al. (2015b)	UV‐H + DMDC (55°C)
Clarified apple juice	Yeasts/Fungi	1.6–3.2/Casco et al. (2025), Gouma et al. (2015a)	3.8 a 5.9/Casco et al. (2025)	UV‐H (mild heat 50°C–60°C)
Turbid apple juice	*E. coli* K12	1.1–2.0/Akgün and Ünlütürk (2017)	3.2 a 4.4/Akgün and Ünlütürk (2017)	Coupled UV‐LED (280/365 nm)
Turbid apple juice	*A. acidoterrestris*	~1.0/Sauceda‐Gálvez et al. (2021)	> 5.0/Sauceda‐Gálvez et al. (2021)	UV‐C with recirculation
Apple surface	*L. monocytogenes*	0.7–1.7/Nicolau‐Lapeña et al. (2022), Adhikari et al. (2015)	2.3 a 2.9/Murray et al. (2018)	UV + Ozone + (AOP)
Apple surface	*E. coli* O157:H7	1.2–3.8/Corrêa (2020), Adhikari et al. (2015)	> 7.0/Ho et al. (2020)	UV + heat (66°C)
Freshcut apple	Native flora/Yeasts	1.3–1.8/Graça et al. (2020), Gouma et al. (2015a)	2.0/Corrêa (2020)	UV‐C + PDI (Curcumin)

When applying fluences of 7.5 and 10 kJ/m^2^, Santo et al. (2016) observed significantly greater reductions of *C. sakazakii* (2–2.4 log CFU/g) in apples, concluding that this technology was more effective than electrolyzed water (AEW/NEW) and sodium hypochlorite (SH). Kim et al. (2021) confirmed that the combination of SAEW, FA, and UVC W‐LED resulted in higher reductions of *S. aureus* (1.65 log CFU/g) and *L. monocytogenes* (2.63 log CFU/g) in cut fruits, demonstrating that this combination enhances disinfection through multiple mechanisms of action against microorganisms, thereby providing greater food safety and quality for fresh‐cut apples. Similarly, Chen et al. (2017) reported that the combined treatment of UV‐C and lactic acid bacteria substantially reduced *Salmonella enteritidis* counts in minimally processed apples, with greater stability compared to control samples.

Food sanitization treatments are essential for ensuring food safety; however, maintaining the cold chain is also indispensable. Santo et al. (2016) and Graça et al. (2020) agreed that storing fresh‐cut fruits at 4°C inhibits the growth of pathogens ( *C. sakazakii* ) and yeasts.

Chen et al. (2016) and Kim et al. (2021) reported that combined treatments (UV‐C + citric acid and UV‐C + electrolyzed water + fumaric acid) significantly reduced browning reactions and maintained higher L* values than control samples, indicating greater brightness or lightness in the treated samples. Chen et al. (2016) attributed the inhibition of browning to the formation of a thin, dry film induced by UV‐C radiation, which acts as a barrier to oxygen. In contrast, Hasan et al. (2017) observed that exposure to UV‐C light caused additional membrane damage, reflected in a slight increase in browning in irradiated apples after three days of storage.

In apple juice, research on UV‐C combined with other technologies has focused primarily on inactivating pathogenic and spoilage microorganisms. This approach acts as a barrier technology to improve food safety and minimize negative impacts on product quality. The main parameters assessed include microbial inactivation kinetics and the influence of intrinsic medium characteristics and pre‐treatment conditions (Gabriel 2015; Gouma et al. 2015a). In turbid apple juice samples, UV‐C technology was significantly less effective than in clarified juice samples, as expected, since suspended particles shield the juice by blocking UV‐C light penetration and reducing radiation efficacy (Sauceda‐Gálvez et al. 2020).

The synergistic lethal effect of UV‐C (2.9 J/mL) and heat results from the inhibition of cellular DNA repair mechanisms, possibly due to heat‐induced fluidization of the cytoplasmic membrane (Gouma et al. 2015a). For *L. monocytogenes* , UV‐C lethality increased synergistically with temperature between 50°C and 60°C, while for *E. coli* and *Salmonella enterica* , maximum synergy occurred between 50°C and 55°C, achieving a 5‐log CFU/mL inactivation of *E. coli* without compromising product quality (Gayán et al. 2015; Gouma et al. 2015a, 2015b). For yeasts ( *Saccharomyces cerevisiae* ), the combined treatment (UV‐C [3.92 J/mL] + heat) showed less pronounced synergy compared to bacteria (Gouma et al. 2015a).

The combination of UV‐C and ultrasound (US) was evaluated for the inactivation of *E. coli* O157:H7 and *A. acidoterrestris* spores. Higher inactivation rates of *E. coli* O157:H7 were observed with the combined treatment compared to the individual technologies. This effect can be attributed to the agitation (cavitation) induced by ultrasound, which increases the exposure of microorganisms to UV‐C in the opaque medium (Gabriel 2015). In contrast, Tremarin et al. (2017a), using the same combination (US + UV‐C) to inactivate *A. acidoterrestris* spores, concluded that the two technologies exhibited additive rather than synergistic effects.

Cavitation is the formation and violent collapse of microbubbles, generating extreme temperature, pressure, and localized mechanical shear. This phenomenon benefits food processing and disinfection by (i) damaging microbial cell membranes, which facilitates the entry of reactive species, and (ii) enhancing the mixing and penetration of substances such as oxygen and phenolic compounds into materials. By disrupting structures and increasing permeability, ultrasound improves the effectiveness of subsequent treatments, such as UV‐C radiation (Lauteri et al. 2023; Char et al. 2011).

Another widely studied combination in the literature is ultra‐high hydrostatic pressure (HHP) (Sauceda‐Gálvez et al. 2021, 2020, 2019). This technology applies pressures between 300 and 600 MPa uniformly throughout the product for a few minutes, resulting in (i) denaturation of enzymatic and structural proteins, (ii) irreversible damage to microbial membranes, and (iii) partial inactivation of oxidative enzymes such as PPO and POD. Unlike conventional heat treatment, HHP preserves aroma, color, and vitamins, as the process involves mild temperatures (generally below 45°C), which prevents the degradation of heat‐sensitive compounds and maintains the sensory and nutritional qualities of the fresh product (Goraya et al. 2024; Oey et al. 2008).

UV‐C interactions for the inactivation of resistant microbial spores were studied, and it was observed that previously applied high pressure (HHP) subtly deformed the cell wall (creating fissures and compressed spines), making the ascospores more vulnerable to damage caused by UV‐C (Sauceda‐Gálvez et al. 2019).

The HHP and UV‐C combination was also evaluated for inactivating *A. acidoterrestris* spores in clarified apple juice samples, and the HHP treatment did not significantly improve inactivation rates. In turbid apple juice samples, HHP altered the matrix properties, increasing turbidity. The increase in opacity caused by HHP apparently reduced the effectiveness of the radiation at the highest dose tested (Sauceda‐Gálvez et al. 2020).

The effects of chemical additives such as dimethyl dicarbonate (DMDC) and natamycin, combined with UV‐C and heat (H), were also evaluated. UV‐C + H (55°C) + DMDC, a potent microbial inhibitor that inactivates cellular enzymes, at concentrations of 25, 50, and 75 mg/L in apple juice for microbial inactivation of *E. coli* , produced a satisfactory synergistic effect. Adding 75 mg/L DMDC before UV‐C/H treatment enabled a 5‐log CFU/mL reduction in just 1.8 min, representing a 44% reduction in processing time and UV‐C dose (Gouma et al. 2015b).

The addition of natamycin, a natural antifungal used to prevent yeast spoilage, at concentrations of 30 ppm or 45 ppm after UV‐C/H treatment (50°C) in apple juice resulted in synergistic inactivation of the yeast cocktail during refrigerated storage. This synergy is attributed to physiological and structural changes induced by UV‐C and heat, such as increased membrane permeability, which accelerate the action of natamycin on cellular components (Casco et al. 2025).

## 4 Conclusions and Future Perspectives

The use of UV‐C radiation on apples and their derivatives has emerged as a promising alternative for microbiological control and maintaining post‐harvest quality. Overall, the reviewed studies indicate that, when applied to fresh fruit, this technology helps reduce or stabilize the microbial load and preserve physicochemical attributes, although its effectiveness depends on the dose and exposure time (Nicolau‐Lapeña et al. 2023; Phonyiam et al. 2021; Adhikari et al. 2015; Pataro et al. 2015).

For minimally processed products, UV‐C is effective in reducing pathogenic microorganisms such as *L. monocytogenes* and *E. coli* and in delaying enzymatic browning without significantly compromising product characteristics. However, the limited penetration of radiation into cut plant tissues or irregular surfaces remains a challenge for large‐scale application (Yuan et al. 2022; Kim et al. 2021; Green et al. 2020; Graça et al. 2020; Chen et al. 2016, 2017).

When applied to apple juices and liquid derivatives, UV‐C is highly effective in reducing bacteria and spores and helps maintain quality during refrigerated storage, thereby extending shelf life (Yang et al. 2019; Akgün and Ünlütürk 2017; Tremarin et al. 2017b; Gabriel 2015). However, the turbidity and opacity of the juice can reduce the effectiveness of the radiation, requiring fluence adjustments or additional strategies.

Overall, the results show that UV‐C can increase microbiological safety, extend shelf life, and preserve the sensory and nutritional characteristics of apples and their derivatives (Yang et al. 2019). Despite these advances, important challenges remain, including the limited penetration of UV‐C radiation, the lack of standardized application protocols, and the need for studies that consider real processing conditions on an industrial scale (Graça et al. 2020; Sauceda‐Gálvez et al. 2021).

For future prospects, it is recommended to expand research in the following areas: (i) economic and environmental assessments to validate the feasibility of large‐scale industrial use, and (ii) international standardization of doses, protocols, and regulations to ensure the technology's effectiveness and safety.

In summary, UV‐C radiation is a promising and sustainable method for sanitizing apples and related products, with the potential to replace or complement chlorine use. It offers a more economically advantageous alternative due to the following factors: (i) Reduced water consumption—UV‐C is a dry disinfection method, eliminating the need for large volumes of water for washing and the associated costs of wastewater disposal and treatment (Graça et al. 2020; Green et al. 2020; Rios de Souza et al. 2020); (ii) Cost‐efficiency for small processors—the technology is considered a low‐cost option, especially attractive to small producers of juices and fresh fruits because of the lower initial investment and simple maintenance (Nicolau‐Lapeña et al. 2022); (iii) Operational input costs—while chlorine requires the ongoing purchase of chemicals and monitoring of toxic residues, UV‐C uses only electrical energy (Graça et al. 2020; Rios de Souza et al. 2020).

However, increased applied research on industrial applications will be essential to establish this technology in the fruit and vegetable industry.

## Author Contributions

**Amauri Rosenthal:** conceptualization, project administration, resources. **Mahyra da Paixão e Silva:** investigation, methodology, validation, visualization, writing – review and editing, formal analysis, writing – original draft. **Caroline Corrêa de Souza Coelho:** investigation, methodology, software, formal analysis, writing – original draft, data curation. **Otniel Freitas‐Silva:** writing – review and editing, conceptualization, methodology, resources, project administration, funding acquisition, writing – original draft, supervision. **Antonio Gomes Soares:** supervision, conceptualization. **Juliana Pereira Rodrigues Belas:** investigation, methodology, validation, visualization, formal analysis, data curation, writing – original draft. **Caroline Rodrigues de Azevedo:** investigation, methodology, validation, visualization, software, formal analysis.

## Funding

This work was supported by Coordenação de Aperfeiçoamento de Pessoal de Nível Superior, 001, Conselho Nacional de Desenvolvimento Científico e Tecnológico (Grant 309528/2025‐9), Instituto Nacional de Ciência e Tecnologia—Frutas Tropicais (Grant 465335/2014‐4), Fundação Carlos Chagas Filho de Amparo à Pesquisa do Estado do Rio de Janeiro (Grants E‐26/201.302/2022 and E‐26/200.270/2026).

## Ethics Statement

The authors have nothing to report.

## Conflicts of Interest

The authors declare no conflicts of interest.

## Supporting information


**Data S1:** fsn372316‐sup‐0001‐Supinfo.docx.

## Data Availability

The data that support the findings of this study are available from the corresponding author upon reasonable request.
