# Supplementary material for: Global Overview of the Use and Effectiveness of Ultraviolet C Technology in Fresh, Minimally Processed Apples and Apple Juice Over the Last 10 Years
Source: Food Sci Nutr. 2026 Sep 17;14(9):e72316. doi: 10.1002/fsn3.72316 (PMC13586447; doi:10.1002/fsn3.72316)
Supplement: Supplementary file 1 [file FSN3-14-e72316-s001.docx]

**SUPPLEMENTARY MATERIAL**

**GLOBAL OVERVIEW OF THE USE AND EFFECTIVENESS OF ULTRAVIOLET C TECHNOLOGY IN FRESH, MINIMALLY PROCESSED APPLES, AND APPLE JUICE OVER THE LAST 10 YEARS**

Mahyra da Paixão e Silva^1^, Caroline Rodrigues de Azevedo^2^, Caroline Corrêa de Souza Coelho^3^, Juliana Pereira Rodrigues Belas^2^, Antonio Gomes Soares^4^, Amauri Rosenthal^4^, Otniel Freitas-Silva^4^

^1^ Universidade Federal Rural do Rio de Janeiro, Depto. de Ciência e Tecnologia de

Alimentos, Rod. BR 465, km 7 – 23890-000 – Seropédica, RJ, Brazil.

^2^ Universidade Federal do Estado do Rio de Janeiro, Av. Pasteur, 296 - Urca, 22290-240, Rio de Janeiro, RJ, Brazil.

^3^ Embrapa Roraima, Av. Brasil, 3911 –69315-292– Boa Vista, RR, Brazil.

^4^ Embrapa Agroindústria de Alimentos, Av. das Américas, 29501 – 23020-470 – Rio

de Janeiro, RJ, Brazil.

**Corresponding author**

E-mail address: otniel.freitas@embrapa.br

Tel.: +55-21-3622-9645

This systematic review was conducted in accordance with the Preferred Reporting Items for Systematic Reviews and Meta-Analyses (PRISMA) guidelines. The review process is described below.

**Planning**

The planning stage involves defining and completing all steps included in the search protocol within the software, which supports the entire search process, selection, and data extraction. All steps are described below:

Objective: To evaluate, based on studies published in the past 10 years, the application of UV-C radiation to fresh and processed apple products and its effects on microbiological, physicochemical, and sensory quality.

Main question: What is the effectiveness of UV-C technology in fresh and processed apple products?

**Use PICOC Criteria**

**Population:** Apple and processed apple products, including minimally processed apples, apple juice (clarified or cloudy), and other apple-derived products.

**Intervention:** Application of UV-C radiation as a non-thermal sanitization treatment under various processing conditions, including radiation dose (fluence), exposure time, and application mode (standalone or combined with other technologies).

**Control:** Conventional sanitization methods or alternative non-thermal technologies.

**Outcomes (Results):** UV-C dose (fluence), exposure time, treatment configuration, microbial inactivation, and changes in quality parameters in apple and processed apple products, including physicochemical attributes, color parameters, levels of bioactive compounds, enzymatic activity, and sensory attributes.

**Context (Application):** Studies conducted in food processing, post-harvest technology, and food safety of apple products.

**Exact Boolean search strings:** (apple OR "*Malus domestica*") AND ("ultraviolet c" OR "UV-C" OR UVC OR "254 nm")

**Source Selection Criteria Definition:**

**Criterion:** Works indexed in journals in the field of Food Science and Technology available in web databases; works published after 2015.

**Languages Studied:** English

**Sources Search Methods:** Titles, abstracts, and keywords were independently screened, followed by the application of predefined inclusion and exclusion criteria. Full-text articles were assessed when eligibility could not be determined from the initial screening.

**Source List:**

**Source:** Science Direct, Scopus and Web of Science

**Study selection criteria (inclusion and exclusion):**

**Criterion of Inclusion:** Studies on fresh apples (in natura); studies on minimally processed apples; studies on apple juice; studies on apple-derived products; studies applying UV-C technology as a stand-alone treatment; studies applying UV-C technology in combination with other preservation methods.

**Exclusion Criteria:** Review articles; studies related to fruits other than apple; studies addressing fruit blends; studies evaluating technologies other than UV-C; studies in which the object of analysis was not apple or apple-derived products.

**Studies Initial Selection:** The methodological quality of the included studies was assessed through a critical appraisal of the study design after the systematic review selection process.

**Results summarization:** After full-text assessment of the included studies, data were systematically synthesized using quantitative and qualitative approaches. Quantitative analysis summarized key variables and outcomes across studies, while qualitative synthesis identified strengths and limitations in the application of UV-C technology. Studies were grouped by relevant categories, including product type (e.g., fresh apples, fresh-cut apples, apple juice, and derivatives), processing conditions, microbiological outcomes, and quality-related parameters. The findings were critically discussed to compare methodologies, processing conditions, and reported effects.

**Execution**

After completing the Protocol, the Execution stage was conducted.

- Studies Identification: The search strings were applied to each database as described below, and the results were exported in RIS format to StArt (State of the Art through Systematic Review, version 3.0.3 BETA):

a) Scopus: (TITLE-ABS-KEY(apple OR "Malus domestica") AND TITLE-ABS-KEY("ultraviolet c" OR "UV-C" OR UVC OR "254nm") AND PUBYEAR > 2014 AND PUBYEAR < 2025 AND (LIMIT-TO(DOCTYPE, "ar")) AND (LIMIT-TO(LANGUAGE, "English"))).

b) Science Direct: Title, abstract, keywords: (apple OR "Malus domestica") AND ("ultraviolet c" OR "UV-C" OR UVC OR "254nm") AND pub-date > 2014 AND pub-date < 2026.

c) Web of Science: TOPIC: (apple OR "Malus domestica") AND TOPIC: ("ultraviolet c" OR "UV-C" OR UVC OR "254nm"). Refined by: DOCUMENT TYPES: (ARTICLE) AND LANGUAGES: (ENGLISH). PUBLICATION YEARS: 2015-2025. Indexes: SCI-EXPANDED, SSCI, A&HCI, CPCI-S, CPCI-SSH, ESCI.

**-Selection:** In the selection stage, studies submitted to the StArt program were evaluated according to the criteria described in the Protocol.

**-Extraction:** All studies accepted after applying the inclusion and exclusion criteria were read in full. In this phase, studies were classified as accepted, rejected, or duplicates. A study was rejected if it was not relevant to answering the main question defined in the protocol. Relevant information addressing the research question was extracted only from studies classified as accepted, using a predefined data extraction form (Supplementary Data), as specified in the study protocol.

**Supplementary Data:**

- Title

- Year of publication

- Countries of publication

- Journal name

- Subject of study

- Sampling

- Technology used (UV-C alone, UV-C combined, applied fluence, exposure time)

- Storage

- Influence of UV-C on quality, chemical, and microbiological parameters

**Summarization**

StArt (State of the Art through Systematic Review, version 3.0.3 BETA), developed by the Software Engineering Research Laboratory (LaPES/UFSCar), was used to support the systematic review process. Data analysis included the creation of graphs and figures using Canva and Google Charts, as well as the construction of bibliometric network maps with VOSviewer (Java version 1.6.19). At this stage, results were synthesized based on the search and refinement of studies, using data extracted from the included studies according to a predefined extraction protocol. The StArt software facilitated the organization and categorization of relevant topics. Additionally, the dataset generated from the extracted studies was exported in Excel format, and further data analysis was conducted using Microsoft Excel.

**Variability in Experimental Reliability**

The discussion on the variability in experimental reliability among selected studies investigating UV-C treatment of fresh, minimally processed apples and apple juice reveals that the efficacy of this technology is influenced by a complex interaction of physical, chemical, and biological factors (AKWU et al., 2023).

The following key points underlie this issue (NICOLAU-LAPEÑA et al., 2022; GREEN et al., 2020; RIOS DE SOUZA et al., 2020; SAUCEDA-GÁLVEZ et al., 2020; SYAMALADEVI et al., 2015):

i) lack of precise optical data and rigorous verification of the applied UV-C dose (fluence);

ii) variability in irradiation sources: low-pressure mercury lamps (LPM) and UVC-LEDs operate at different wavelengths and therefore exhibit different germicidal efficacies depending on the absorption peak of the target microorganism’s DNA;

iii) variation among strains: different strains of the same species exhibit varying levels of resistance to UV-C, which explains discrepancies in the decimal reduction dose (D10) values reported in the literature;

iv) physiological state of the microorganism: the history of the cells (such as previous exposure to suboptimal growth conditions like acidity or desiccation) can induce heterologous resistance responses, making microorganisms more robust to subsequent UV-C treatment;

v) standardization of sample preparation: methodological differences in matrix preparation can also lead to variability in whole fruits (since surface morphology – such as roughness, presence of waxes, or microcracks – can create a shading effect, shielding pathogens from radiation) and in juices (turbidity drastically alters the efficiency of UV-C due to light scattering by suspended solids).

In conclusion, despite the intrinsic variability caused by the distinct optical properties of each matrix (such as turbidity and absorption coefficients) and the diversity of applied fluences, there is substantial scientific reliability demonstrated by the consistent results across studies. This reliability is supported by three main pillars identified in the literature (MENEZES et al., 2020; MENEZES et al., 2019; GOUMA et al., 2015a):

**i) Rigor in Mathematical Modeling:** Most studies show high statistical accuracy, with coefficients of determination (R²) often exceeding 0.90 and low root mean square errors (RMSE). The use of nonlinear models, such as Weibull and Biphasic, has enabled various authors to accurately describe inactivation kinetics, validating the efficacy of radiation even in complex media

**ii) Reproducibility of Microbial Efficacy:** Regardless of the device used (static chambers, flow reactors, or LEDs), studies consistently confirm that UV-C achieves the safety target of a 5-log reduction for critical pathogens such as E. coli and L. monocytogenes, provided the dose is correctly calculated and delivered. Furthermore, the relative sensitivity of microorganisms (such as the greater resistance of fungal spores compared to vegetative bacteria) shows a consistent pattern throughout the literature reviewed

**iii) Stability of Quality Parameters:** The literature consistently shows that UV-C radiation, unlike thermal treatments, preserves the essential physicochemical characteristics (Brix, pH, and acidity) of apple juice, reinforcing the reliability of this technology in maintaining the “fresh product” standard required by the industry.

Therefore, the reported variability should not be interpreted as a lack of precision, but rather as a necessary reflection of technological adaptation to the specific characteristics of each matrix. Consistency in microbial inactivation and retention of bioactive compounds establishes UV-C as a safe, predictable, and technically mature tool for processing apple-derived products (CASCO et al., 2025; AKWU et al., 2023).

**REFERENCES**

ABPM. Brazilian Apple Producers Association. Available at: www.abpm.org.br.

ADHIKARI, A. *et al.* Ultraviolet-C light inactivation of *Escherichia coli O157: H7* and *Listeria monocytogenes* on organic fruit surfaces. International Journal of Food Microbiology, [*s. l.*], vol. 210, p. 136–142, 2015.

AKGÜN, M. P.; ÜNLÜTÜRK, S. Effects of ultraviolet light emitting diodes (LEDs) on microbial and enzyme inactivation of apple juice. International Journal of Food Microbiology, [*s. l.*], vol. 260, p. 65–74, 2017.

AKWU, A. S. Effect of germicidal short wave-length ultraviolet light on the polyphenols, vitamins, and microbial inactivation in highly opaque apple juice. Journal of Food Safety, vol. 43, no. 3, 13037. 2023.

CASCO, M. de los A. *et al.* A novel approach using UV-C light assisted by mild heat and natamycin as a hurdle strategy to preserve juice from non-commercial-grade apples. Food Control, [*s. l.*], vol. 171, 2025.

CHEN, C. et al. Combined treatment of ultraviolet-C and *L. plantarum* on *Salmonella enteritidis* and quality control of fresh-cut apple. Journal of Food Processing and Preservation, https://doi.org/10.1111/jfpp.13349. 2017.

CHEN, C. *et al.* Effect of citric acid combined with UV-C on the quality of fresh-cut apples. Postharvest Biology and Technology, [*s. l.*], vol. 111, p. 126–131, 2016.

CORRÊA, T. Q. Effects of ultraviolet light and curcumin-mediated photodynamic T inactivation on microbiological food safety: A study in meat and fruit. Photodiagnosis and Photodynamic Therapy, vol. 30, 101678. 2020.

CORSON, E. et al. Hepatitis A virus inactivation in phosphate buffered saline, apple juice and coconut water by 254 nm and 279 nm ultraviolet light systems. Food Microbiology, vol. 129, 104756. 2025

DIAS, T. G. Physicochemical characterization, antioxidant activity and total phenolic content in 'Gala' apples subjected to different UV-C radiation doses. Acta Scientiarum, vol. 39, no. 1, p. 67-73. 2017.

EUROPEAN PARLIAMENT AND THE COUNCIL OF THE EUROPEAN UNION. 1999.

FAOSTAT. Food and Agriculture Organization. Available at: www.fao.org.

FORTIN, N.D. Chapter 11: Global governance of food safety: the role of the FAO, WHO and Codex Alimentarus in regulatory harmonization. Research Handbook on International Food Law, p. 227-242, 2023.

GABRIEL, A. A. Combinations of selected physical and chemical hurdles to inactivate Escherichia coli O157: H7 in apple and orange juices. Food Control, [*s. l.*], vol. 50, p. 722–728, 2015.

GABRIEL, A. A.; ANCOG, M. M. L. Effects of suboptimal growth conditions on the subsequent UV-C resistance of *Listeria monocytogenes* in coconut liquid endosperm and apple juice. LWT, [*s. l.*], vol. 99, p. 460–467, 2019.

GAYÁN, E. *et al.* Environmental and biological factors influencing the UV-C resistance of *Listeria monocytogenes*. Food Microbiology, [*s. l.*], vol. 46, p. 246–253, 2015.

GOK, S. B. UV-C Treatment of Apple and Grape Juices by Modified UV-C Reactor Based on Dean Vortex Technology: Microbial, Physicochemical and Sensorial Parameters Evaluation. Food and Bioprocess Technology, vol. 14, p. 1055–1066. 2021.

GÓMEZ, P. L. *et al.* Potential of UV-C Light for Preservation of Cut Apples Fortified with Calcium: Assessment of Optical and Rheological Properties and Native Flora Dynamics. Food and Bioprocess Technology, [*s. l.*], vol. 8, n^o^ 9, p. 1890–1903, 2015.

GOUMA, M. *et al.* Inactivation of spoilage yeasts in apple juice by UV-C light and in combination with mild heat. Innovative Food Science and Emerging Technologies, [*s. l.*], vol. 32, p. 146–155, 2015a.

GOUMA, M. *et al.* Influence of dimethyl dicarbonate on the resistance of Escherichia coli to a combined UV-Heat treatment in apple juice. Frontiers in Microbiology, [*s. l.*], vol. 6, n^o^ MAY, 2015b.

GOUMA, M. Modelling microbial inactivation kinetics of combined UV-H treatments in apple juice. Innovative Food Science & Emerging Technologies, vol. 27, p. 111-120. 2015c.

GRAÇA, A. *et al.* The effect of UV-C and electrolyzed water on yeasts on fresh-cut apple at 4 °C. Journal of Food Engineering, [*s. l.*], vol. 282, 2020.

GREEN, A. *et al.* The efficacy of UVC LEDs and low pressure mercury lamps for the reduction of *Escherichia coli O157:H7* and *Listeria monocytogenes* on produce. Innovative Food Science and Emerging Technologies, [*s. l.*], vol. 64, 2020.

GUO et al. Efficacy and mechanism of ultrasound combined with slightly acidic electrolyzed water for inactivating *Escherichia coli*. Journal of Food Quality, vol. 1, 6689751, p. 1-10. 2021.

HO, J. *et al.* Degradation of chlorpyrifos and inactivation of *Escherichia coli O157:H7* and *Aspergillus niger* on apples using an advanced oxidation process. Food Control, [*s. l.*], vol. 109, 2020.

HUSSAIN, M.S., TANGO, C.N., OH, D.H. Inactivation kinetics of slightly acidic electrolyzed water combined with benzalkonium chloride and mild heat treatment on vegetative cells, spores and biofilms of Bacillus cereus. Food Research International, vol. 116, p. 157-167. 2019.

KIM, G. H.; LEE, C. L.; YOON, K. S. Combined hurdle technologies using UVC waterproof LED for inactivating foodborne pathogens on fresh-cut fruits. Foods, [*s. l.*], vol. 10, n^o^ 8, 2021.

KEENER, L.; KOUTCHMA, T. Chapter 27 – Capacity building: Harmonization and achieving food safety in an era of unilateral legislation. Ensuring Global Food Safety (second edition), p. 489-502, 2022.

ISLAM, M. S. et al. Effect of UV Irradiation on the Nutritional Quality and Cytotoxicity of Apple Juice. Journal of Agricultural and Food Chemistry, vol. 64, p. 7812−7822. 2016a.

ISLAM, M. S. et al. UV-C irradiation as an alternative disinfection technique: Study of its effect on polyphenols and antioxidant activity of apple juice. Innovative Food Science and Emerging Technologies, vol. 34, p. 344–351. 2016b.

LUCIANO-ROSARIO, D. et al. Mold in, mold out: Harvest bins harbor viable inoculum that can be reduced using novel sanitation methods to manage blue mold decay of apples. Postharvest Biology and Technology, vol. 221, 113323. 2025.

MENEZES, N. M. C. et al. Effect of soluble solids concentration on *Neosartorya fischeri* inactivation using UV-C light. International Journal of Food Microbiology, vol. 296, p. 43–47. 2019.

MENEZES, N. M. C. et al. Modeling the inactivation of *Aspergillus fischeri* and *Paecilomyces niveus* T ascospores in apple juice by different ultraviolet light irradiances. International Journal of Food Microbiology, vol. 333, 108773. 2020.

MURRAY, K. *et al.* Inactivation of listeria monocytogenes on and within apples destined for caramel apple production by using sequential forced air ozone gas followed by a continuous advanced oxidative process treatment. Journal of Food Protection, [*s. l.*], vol. 81, n^o^ 3, p. 357–364, 2018.

NICOLAU-LAPEÑA, I. *et al.* Ultraviolet Applications to Control Patulin Produced by *Penicillium expansum* CMP-1 in Apple Products and Study of Further Patulin Degradation Products Formation and Toxicity. Food and Bioprocess Technology, [*s. l.*], vol. 16, n^o^ 4, p. 804–823, 2023.

NICOLAU-LAPEÑA, I. *et al.* Inactivation of *Escherichia coli, Salmonella enterica* and *Listeria monocytogenes* on apple peel and apple juice by ultraviolet C light treatments with two irradiation devices. International Journal of Food Microbiology, [*s. l.*], vol. 364, 2022.

NGNITCHO et al. Inactivation of bacterial pathogens on lettuce, sprouts and spinach using hurdle technology. Innovative Food Science and Emerging Technologies, vol. 43, p. 68-76. 2017.

ORLOWSKA, M. et al. Surrogate organisms for pathogenic O157:H7 and non-O157 *Escherichia coli* strains for apple juice treatments by UV-C light at three monochromatic wavelengths. Food Control, vol. 47, 647e655. 2015.

PATARO, G.; DONSI, G.; FERRARI, G. Post-harvest UV-C and PL irradiation of fruits and vegetables. Chemical Engineering Transactions, [*s. l.*], vol. 44, p. 31–36, 2015.

PÉREZ-LORENZO, E. et al. Reduction of lactic acid bacteria and acetic acid bacteria from natural apple cider by UVC irradiation. Journal of Food Processing and Preservation, vol. 46, no. 11, e17136. 2022.

PHONYIAM, O. *et al.* UV-C irradiation before or after inoculation with *Colletotrichum gloeosporioides* affected phytohormones and antioxidants in apple fruit (Malus domestica Borkh.). European Journal of Horticultural Science, [*s. l.*], vol. 86, n^o^ 6, p. 620–628, 2021.

RIOS DE SOUZA, V. *et al.* A comparative study on the inactivation of *Penicillium expansum* spores on apple using light emitting diodes at 277 nm and a low-pressure mercury lamp at 253.7 nm. Food Control, [*s. l.*], vol. 110, 2020.

SANTO, D. *et al.* Survival and growth of *Cronobacter sakazakii* on fresh-cut fruit and the effect of UV-C illumination and electrolyzed water in the reduction of its population. International Journal of Food Microbiology, [*s. l.*], vol. 231, p. 10–15, 2016.

SAUCEDA-GÁLVEZ, J. N. *et al.* Short wave ultraviolet light (UV-C) effectiveness in the inactivation of bacterial spores inoculated in turbid suspensions and in cloudy apple juice. Beverages, [*s. l.*], vol. 7, n^o^ 1, 2021.

SAUCEDA-GÁLVEZ, J. N. *et al.* Effect of single and combined UV-C and ultra-high pressure homogenisation treatments on inactivation of *Alicyclobacillus acidoterrestris* spores in apple juice. Innovative Food Science and Emerging Technologies, [*s. l.*], vol. 60, 2020.

SAUCEDA-GÁLVEZ, J. N. *et al.* Inactivation of ascospores of *Talaromyces macrosporus* and *Neosartorya spinosa* by UV-C, UHPH and their combination in clarified apple juice. Food Control, [*s. l.*], vol. 98, p. 120–125, 2019.

SHIN, M. *et al.* Application of combined treatment of peracetic acid and ultraviolet-C for inactivating pathogens in water and on surface of apples. International Journal of Food Microbiology, [*s. l.*], vol. 411, 2024.

SMEDT, K.; VOS, E. The application of the Precautionary Principle in the EU. The Responsibility of Science, vol. 57, p. 163-186, 2022.

SYAMALADEVI, R. M. et al. Ultraviolet-C light inactivation of *Penicillium expansum* on fruit surfaces. Food Control, vol. 50, 297e303. 2015.

TCHONKOUANG, R. D.; LIMA, A. R.; QUINTINO, A. C.; CRISTOFOLI, N. L.; VIEIRA, M. C. UV-C light: A Promising Preservation Technology for Vegetable-Based Nonsolid Food Products. Foods, vol. 12 (17), 3227, 2023.

TIRONO, M. The Effectiveness of UV-C Light for Inactivating *Listeria monocytogenes* Bacteria and its Impact on Apple Juice. The Open Biotechnology Journal, [*s. l.*], vol. 17, n^o^ 1, 2023.

TREMARIN, A.; BRANDÃO, T. R. S.; SILVA, C. L. M. Application of ultraviolet radiation and ultrasound treatments for *Alicyclobacillus acidoterrestris* spores inactivation in apple juice. LWT, [*s. l.*], vol. 78, p. 138–142, 2017a.

TREMARIN, A.; BRANDÃO, T. R. S.; SILVA, C. L. M. Inactivation kinetics of *Alicyclobacillus acidoterrestris* in apple juice submitted to ultraviolet radiation. Food Control, [*s. l.*], vol. 73, p. 18–23, 2017b.

YANG, Y. *et al.* Effect of thermal pasteurization and ultraviolet treatment on the quality parameters of not-from-concentrate apple juice from different varieties. CYTA - Journal of Food, [*s. l.*], vol. 17, n^o^ 1, p. 189–198, 2019.

YIN, F. et al. Inactivation and potential reactivation of pathogenic *Escherichia coli* *O157:H7* in apple juice following ultraviolet light exposure at three monochromatic wavelengths. Food Microbiology, vol. 46, 329e335. 2015.

XIANG, Q. et al. Effect of UVC light-emitting diodes on apple juice: Inactivation of T *Zygosaccharomyces rouxii* and determination of quality. Food Control, vol. 111, 107082. 2020.
